# Coiled-coil protein composition of 22 proteomes – differences and common themes in subcellular infrastructure and traffic control

**DOI:** 10.1186/1471-2148-5-66

**Published:** 2005-11-16

**Authors:** Annkatrin Rose, Shannon J Schraegle, Eric A Stahlberg, Iris Meier

**Affiliations:** 1 Department of Plant Cellular and Molecular Biology, Plant Biotechnology Center, Ohio State University, 1060 Carmack Road, Columbus, OH 43210, USA; 2 Ohio Super Computer Center, 1224 Kinnear Road, Columbus, OH 43212, USA

## Abstract

**Background:**

Long alpha-helical coiled-coil proteins are involved in diverse organizational and regulatory processes in eukaryotic cells. They provide cables and networks in the cyto- and nucleoskeleton, molecular scaffolds that organize membrane systems and tissues, motors, levers, rotating arms, and possibly springs. Mutations in long coiled-coil proteins have been implemented in a growing number of human diseases. Using the coiled-coil prediction program MultiCoil, we have previously identified all long coiled-coil proteins from the model plant *Arabidopsis thaliana *and have established a searchable Arabidopsis coiled-coil protein database.

**Results:**

Here, we have identified all proteins with long coiled-coil domains from 21 additional fully sequenced genomes. Because regions predicted to form coiled-coils interfere with sequence homology determination, we have developed a sequence comparison and clustering strategy based on masking predicted coiled-coil domains. Comparing and grouping all long coiled-coil proteins from 22 genomes, the kingdom-specificity of coiled-coil protein families was determined. At the same time, a number of proteins with unknown function could be grouped with already characterized proteins from other organisms.

**Conclusion:**

MultiCoil predicts proteins with extended coiled-coil domains (more than 250 amino acids) to be largely absent from bacterial genomes, but present in archaea and eukaryotes. The structural maintenance of chromosomes proteins and their relatives are the only long coiled-coil protein family clearly conserved throughout all kingdoms, indicating their ancient nature. Motor proteins, membrane tethering and vesicle transport proteins are the dominant eukaryote-specific long coiled-coil proteins, suggesting that coiled-coil proteins have gained functions in the increasingly complex processes of subcellular infrastructure maintenance and trafficking control of the eukaryotic cell.

## Background

The coiled-coil was one of the earliest protein structures described and first discovered in the two-stranded coiled-coil protein alpha-keratin [1]. Coiled-coils consist of two or more alpha-helices winding around each other in a supercoil, a simple yet versatile protein fold [2]. Mutations in coiled-coil proteins have been implicated in a large variety of human diseases such as severe skin fragility, muscular dystrophies, neurodegenerative diseases, progeria, and cancer [3-10]. Spurred by medical interest, the number of investigated long coiled-coil proteins in yeast and animals has rapidly grown in recent years. Recently, a database of all long coiled-coil proteins in the model plant Arabidopsis was established to facilitate the identification and characterization of long coiled-coil proteins in plants [11]. In contrast to eukaryotic organisms, only few long coiled-coil proteins have been characterized in prokaryotes. Examples include chaperonins and nucleases, secretion proteins, and cytadherence factors [12-15].

The foremost feature of coiled-coil domains appears to be their ability to act as "cellular velcro" to hold together molecules, subcellular structures, and even tissues. They can act as protein-protein interaction motifs, for examples as dimerization domains in transcription factors and receptor kinases [16-18]. They function as "zippers" in membrane fusion proteins [19], and as adapters between molecules and solid state cellular structures, such as in microtubule organizing centers, the nuclear pores and lamina, actin- and microtubule-associated proteins and cytoskeleton-associated E3 ubiquitin ligases [20-24]. Extracellular coiled-coil proteins include cell adherence factors and surface receptors, vertebrate blood components such as apolipoproteins and fibrinogen-like clotting factors, and extracellular matrix components such as laminins and cartilage matrix proteins forming tissue scaffolds in metazoa [25,26].

Besides associating with and interconnecting other molecules and macromolecular structures, long coiled-coil domains exhibit a number of structural and mechanical functions [27]. Typically, long coiled-coil domains form rod-like tertiary structures [2] and assemble to dynamic fibers, meshworks and scaffolds. Examples are the intermediate filaments of the cytoskeleton and nuclear lamina [28]. Recent evidence suggests an important role for the dynamic properties of cytoplasmic intermediate filaments in neurodegenerative diseases [29]. Other coiled-coils act as spacers, for example in the yeast spindle pole body where the distance between the plaques is determined by the length of the coiled-coil domain in the connecting proteins [30,31]. Membrane-bound coiled-coil proteins such as the spectrins and golgins form scaffolds for membrane structures within the cell [32,33]. In combination with other functional domains, coiled-coil domains are an integral part of molecular motors, such as the actin motor myosin and the microtubule motors kinesin and dynein [34]. Other coiled-coil proteins with ATPase and GTPase domains often function in folding and repair, e.g. as chaperonins in protein folding, and topoisomerases and nucleases in DNA remodeling [35-37].

On a primary structure level, amino acid sequences with the capacity to form left-handed alpha-helical coiled-coils are characterized by a heptad repeat pattern in which residues in the first and fourth position are hydrophobic, and residues in the fifth and seventh position are predominantly charged or polar [38]. This pattern of hydrophobic and polar residues interferes with sequence comparison algorithms, which often lead to false predictions of homology between long coiled-coil proteins based on the low complexity and repeat nature of the underlying sequence motif. On the other hand, this repeat pattern can also be used to predict coiled-coil domains in amino acid sequences by computational means [39-42].

In the post-genomics era, such structure-prediction algorithms can now be applied to whole proteomes. Based on the prediction algorithm COILS, roughly 10% of all proteins encoded by eukaryotic genomes contain coiled-coil domains whereas prokaryotic genomes contain only 4–5% [43]. Using the MultiCoil program, one in every 11 proteins in yeast was predicted to contain a coiled-coil sequence [44]. However, these studies did not use a cut-off for domain length to determine coiled-coils. A minimum length of three to four heptad repeats is required for the formation of a stable coiled-coil using synthetic peptides [45-47]. Using this minimum domain length of 20 amino acids (or about three heptad repeats), 5.6% of the predicted ORFs in the fully sequenced Arabidopsis genome were found to encode coiled-coil proteins [11].

In a comparative genomics approach, we determined the coiled-coil content of 22 predicted whole proteomes using the prediction pipeline and processing software developed to create the ARABI-COIL database [11]. The 22 genomes analyzed included four archaeal genomes, ten bacterial genomes (three gram-positive and seven gram-negative species), and eight eukaryotic genomes (two each for yeasts, invertebrates, mammals, and plants).

## Results

Prediction and selection of coiled-coil proteins was performed using the MultiCoil algorithm [42] and the ExtractProp processing software [11]. For the purpose of this study, "long coiled-coil" proteins were defined according to the parameters used to establish the ARABI-COIL database and included all sequences with at least one coiled-coil domain and minimum domain length of 70, two domains and minimum domain length of 50, and three or more domains and minimum domain length of 30 [11].

### Eukaryotic genomes contain higher percentages of long coiled-coil proteins than prokaryotic genomes

**Figure 1:**
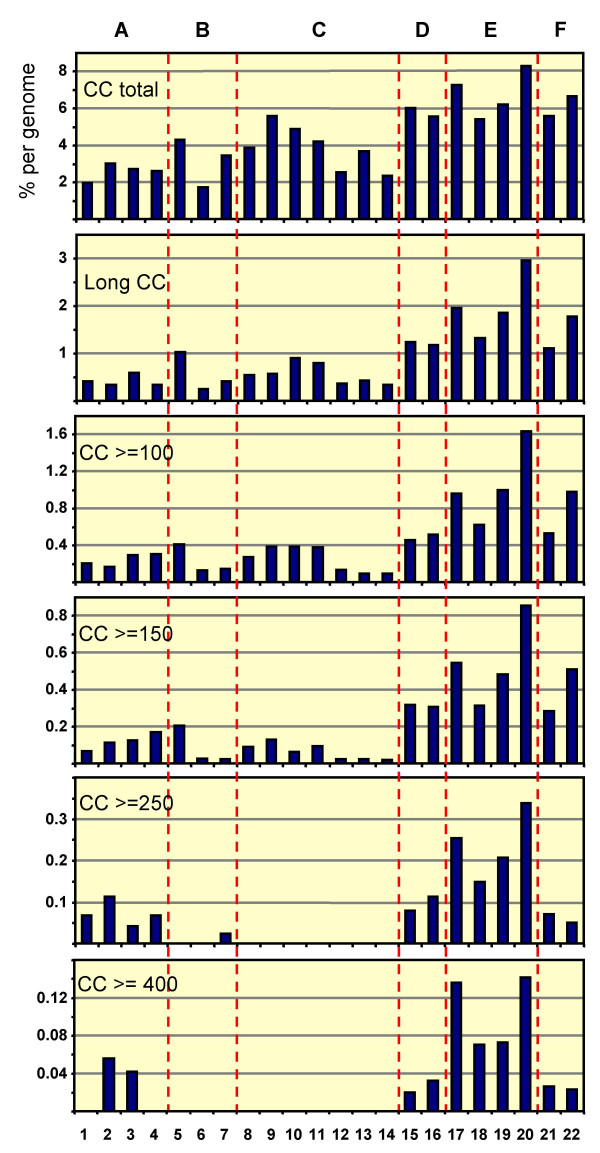
**Percentages of long coiled-coil proteins per genome**. CC, coiled-coil length in amino acids, "CC total" includes all sequences predicted to contain a minimum stretch of 20 amino acids predicted to form a coiled-coil, "Long CC" includes all sequences with at least one coiled-coil domain and minimum domain length of 70, two domains and minimum domain length of 50, and three or more domains and minimum domain length of 30. A, archaea; B, Gram^+ ^bacteria; C, Gram^- ^bacteria; D, yeasts; E, metazoa; F, plants. 1, *Thermoplasma acidophilum*; 2, *Methanococcus jannaschii*; 3, *Archaeoglobus fulgidus*; 4, *Sulfolobus solfataricus*; 5, *Mycoplasma genitalium*; 6, *Mycobacterium tuberculosis*; 7, *Bacillus subtilis*; 8, *Clamydia pneumoniae*; 9, *Heliobacter pylori*; 10, *Borrelia burgdorferi*; 11, *Synechocystis sp. PCC6803*; 12, *Escherichia coli*; 13, *Chromobacterium violaceum*; 14, *Agrobacterium tumefaciens*; 15, *Schizosaccharomyces pombe*; 16, *Saccharomyces cerevisiae*; 17, *Drosophila melanogaster*; 18, *Caenorhabditis elegans*; 19, *Mus musculus*; 20, *Homo sapiens*; 21, *Arabidopsis thaliana*; 22, *Oryza sativa*.

**Figure 2:**
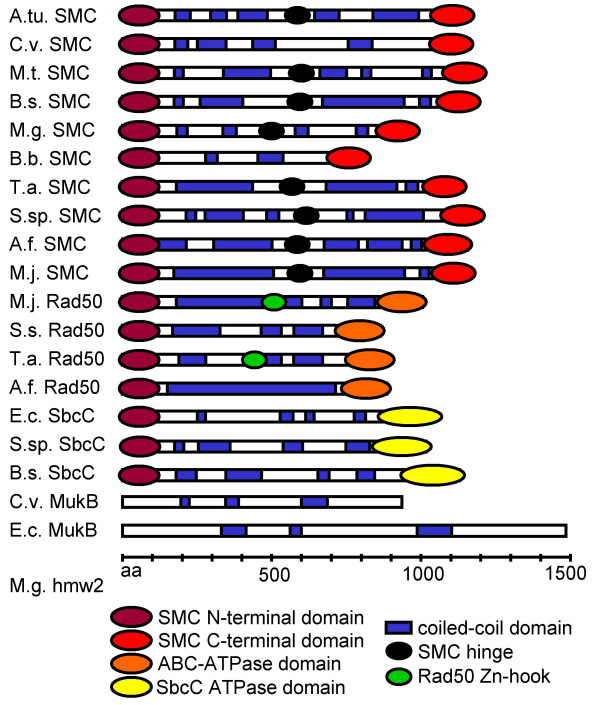
**ABC-ATPases in archaea and bacteria**. Phylogenetic tree and schematic representation of domain structures of ABC-ATPases and related sequences found in the prokaryotic genomes analyzed. Conserved domains shown as identified in CDD [49]. aa, amino acids. For species name abbreviations, see Table 1.

Proteins predicted to form coiled-coil domains were present in all genomes analyzed (Table 1, Figure 1) and comprised between 2% and 8% of the total proteomes. The most pronounced difference between prokaryotic and eukaryotic genomes was in the percentage of genes per genome predicted to encode long or multiple coiled-coil domains. With increasing coiled-coil domain length cut-off, lower percentages of proteins were identified in bacterial genomes. With the exception of *Bacillus subtilis*, MultiCoil predicted no coiled-coil proteins with domains longer than 250 amino acids in the bacterial genomes analyzed. However, archaeal and eukaryotic genomes contain proteins predicted to form coiled-coils of this length. Strikingly, prediction of coiled-coil domains over 400 amino acids in length was completely absent in bacterial genomes, but present in eukaryotes as well as two archaea, *Sulfolobus solfataricus *and *Archeoglobus fulgidus*. These numbers however do not take discontinuous coiled-coil prediction into account, as evident in the case of prokaryotic SMC proteins (Figure 2). 

**Table 1:** Proteome sequence data sets downloaded for MultiCoil analysis

**Organism**	**Number of proteins**	**Date of download**	**Comments (info as provided by EBI and TIGR)**
Archaea (extremophiles):			
*Archaeoglobus fulgidus (A.f.)*	2400	10-Jun-2004	hyperthermophilic, organoheterotrophic-lithoautotrophic, sulfur-metabolizing; glycoprotein envelope, flagellated
*Methanococcus jannaschii (M.j.)*	1782	10-Jun-2004	thermophilic, methanogenic, autotrophic, strict anaerobic; grows under high pressure in deep sea, flagellated
*Sulfolobus solfataricus (S.s.)*	2939	28-Jan-2004	thermophilic, sulfuric acid-producing, aerobic; no flagella, but pilus-like and pseudopodium-like structures
*Thermoplasma acidophilum (T.a.)*	1479	28-Jan-2004	thermoacidophilic; flagellated, no cell wall

Gram-positive bacteria:			
Actinobacteria: *Mycobacterium tuberculosis (M.t.)*	3995	10-Jun-2004	animal pathogen (tuberculosis); no flagella
Bacilli: *Bacillus subtilis (B.s.)*	4167	28-Jan-2004	capable of producing endospores, flagellated
Mollicutes: *Mycoplasma genitalium (M.g.)*	486	10-Jun-2004	animal pathogen (surface parasite), smallest known self-replication cell & genome; no flagella

Gram-negative bacteria:			
Alphaproteobacteria: *Agrobacterium tumefaciens (A.tu.)*	5393	10-Jun-2004	plant pathogen (crown gall); flagellated
Betaproteobacteria: *Chromobacterium violaceum (C.v.)*	4400	10-Jun-2004	subtropical/tropical; produces antimicrobial violacein, flagellated
Gammaproteobacteria: *Escherichia coli K12 (E.c.)*	4356	28-Jan-2004	enterobacterium, laboratory strain, flagellated
Epsilonproteobacteria: *Heliobacter pylori (H.p.)*	1556	10-Jun-2004	animal pathogen; micro-aerophilic, spiral-shaped, flagellated
Chlamydiae: *Chlamydia pneumoniae (C.p.)*	1110	10-Jun-2004	animal pathogen (obligate intracellular parasite), no flagella
Spirochaetes: *Borrelia burgdorferi (B.b.)*	1558	10-Jun-2004	animal pathogen (Lime disease); spiral-shaped, flagellated
Cyanobacteria: *Synechocystis sp. PCC6803 (S.sp.)*	3164	28-Jan-2004	photosynthetic (oxygenic), no flagella

Yeast:			
*Saccharomyces cerevisiae (S.c.)*	6191	28-Jan-2004	baker's yeast
*Schizosaccaromyces pombe (S.p.)*	5037	28-Jan-2004	fission yeast

Metazoa:			
*Caenorhabditis elegans (C.e.)*	22873	28-Jan-2004	nematode
*Drosophila melanogaster (D.m.)*	16196	28-Jan-2004	insect (fruitfly)
*Mus musculus (M.m.)*	27577	28-Jan-2004	mammal (mouse)
*Homo sapiens (H.s.)*	29024	28-Jan-2004	mammal (human)

Plants:			
*Arabidopsis thaliana (A.t.)*	26945	28-Jan-2004	dicot
*Oryza sativa ssp. Japonica (O.s.)*	56056	9-Jan-2004	monocot (rice)

Proteome sequence sets were downloaded from the European Bioinformatics Institute (EBI) [106] or The Institute for Genome Research (TIGR) [107]. The number of protein sequence entries reflects the annotation of ORFs at the time of download.

### Prokaryotic long coiled-coil proteins

#### Archaea

Four archaeal genomes were included in this study and tables with coiled-coil protein details are available in additional file 1 (*Archeoglobus fulgidus*, Table S1; *Methanococcus jannaschii*, Table S2; *Sulfolobus solfataricus*, Table S3; and *Thermoplasma acidophilum*, Table S4). 2–3% of the genes in these archaea were found to code for coiled-coil proteins. In contrast to eubacteria, all of the coiled-coil size-classes analyzed are represented in this group, with proteins predicted to form coiled-coils longer than 400 residues present in *Methanococcus jannaschii *and *Archeoglobus fulgidus *proteomes (see Figure 1).

#### Eubacteria

Bacterial genomes for this study were chosen from different families to represent a wide range of prokaryotic species. Three gram-positive bacterial genomes (additional file 1; *Mycobacterium tuberculosis*, Table S5; *Bacillus subtilis*, Table S6; and *Mycoplasma genitalium*, Table S7), and seven gram-negative bacterial genomes (*Agrobacterium tumefaciens*, Table S8; *Chromobacterium violaceum*, Table S9; *Escherichia coli*, Table S10; *Heliobacter pylori*, Table S11; *Chlamydia pneumoniae*, Table S12; *Borrelia burgdorferi*, Table S13; and the cyanobacterium *Synechocystis*, Table S14) were analyzed.

**Figure 3:**
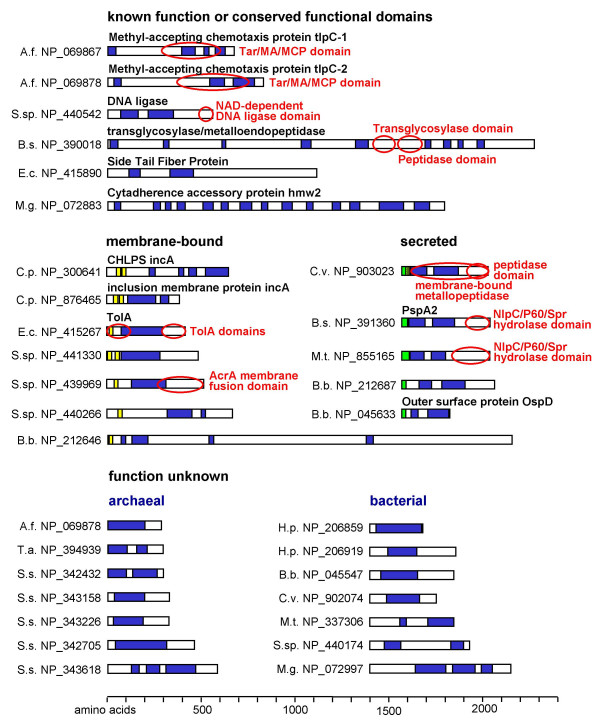
**Prokaryotic long coiled-coil proteins**. Schematic representation of prokaryotic long coiled-coil proteins not belonging to the ABC-ATPase family. Only proteins with at least 150 amino acids predicted to be in a coiled-coil are shown. Blue, coiled-coil domain; green, signal peptide; yellow, transmembrane domain. Functional domains as identified in the CDD [49] are circled in red. tlpC-1, tlpC-2, methyl-accepting chemotaxis proteins homologous to *B.s*. tlpC [111]; hmw2, cytadherence protein [15], CHLPS incA, incA, inclusion membrane proteins [53]; TolA, [52]; OspD, outer surface protein D [112], [113]. For species name abbreviations, see Table 1.

The largest prokaryotic coiled-coil domains were identified in proteins of the SMC, Rad50, SbcC and MukB families. These proteins contain globular head and tail domains separated by a coiled-coil rod with a hinge [48]. Figure 2 summarizes schematic diagrams of the domain structures of the prokaryotic SMC and SMC-like proteins identified in this study based on our coiled-coil prediction data and conserved domains as identified through Conserved Domain Database (CDD) searches [49]. Figure 3 shows a summary of additional long coiled-coil proteins with domains of at least 150 amino acids in length present in prokaryotic genomes. A number of these proteins are involved in membrane events, such as chemosensing via methyl-accepting chemotaxis proteins [50] and membrane fusion and vesicle formation mediated by AcrA, TolA, and incA proteins [51-53]. Others function as adhesion proteins, for example the lambda phage side tail fiber protein [54] and the hmw2 protein of the attachment organelle of *Mycoplasma pneumoniae *[15], or as enzymes of the cell wall such as the NlpC/P60 proteins [55].

### Long coiled-coil domains cause clustering of unrelated coiled-coil sequences

Sequences predicted to form long coiled-coil domains were analyzed for family relationships and conservation across species in an all-against-all approach using the Smith-Waterman sequence comparison algorithm followed by clustering based on an adaptation of Kruskal's minimum cost spanning tree algorithm [56,57].

In a pilot analysis to test the feasibility of the clustering approach, all prokaryotic sequences meeting the aforementioned criteria for "long coiled-coil" proteins were included in the clustering. Due to the larger number of qualified sequences in the eukaryotic species, only the longest domains (at least 250 residues in length) or sequences largely covered by coiled-coil (at least 60% of the sequence) were included in the combined pilot sequence set comprising 527 unique sequences. A maximum P-score of 1.0e-20 was used as the critical threshold when selecting only the most prominent sequence similarities in this test group. In all, 12,013 pair-wise P-score values were selected, defining as many unique relationships from the 277,729 possible pair-wise relationships. Sequences were then grouped using Kruskal's minimum cost spanning tree algorithm using the P-score value as the edge weight for the selected P-score values. 166 independent non-overlapping sequence subsets (subtrees) were defined in this manner. The largest grouping consisted of 270 sequences, representing over half of the sequences in the pilot sequence set and including functionally distinct families such as for example myosins, golgins, and SMC proteins. Distinct clusters of long coiled-coil proteins besides this large, heterogeneous group were formed by the animal and yeast tropomyosins (two separate clusters), the laminins, the CASP/CDP-family and the nuclear lamins.

### Masking of coiled-coil domains before clustering

**Figure 4:**
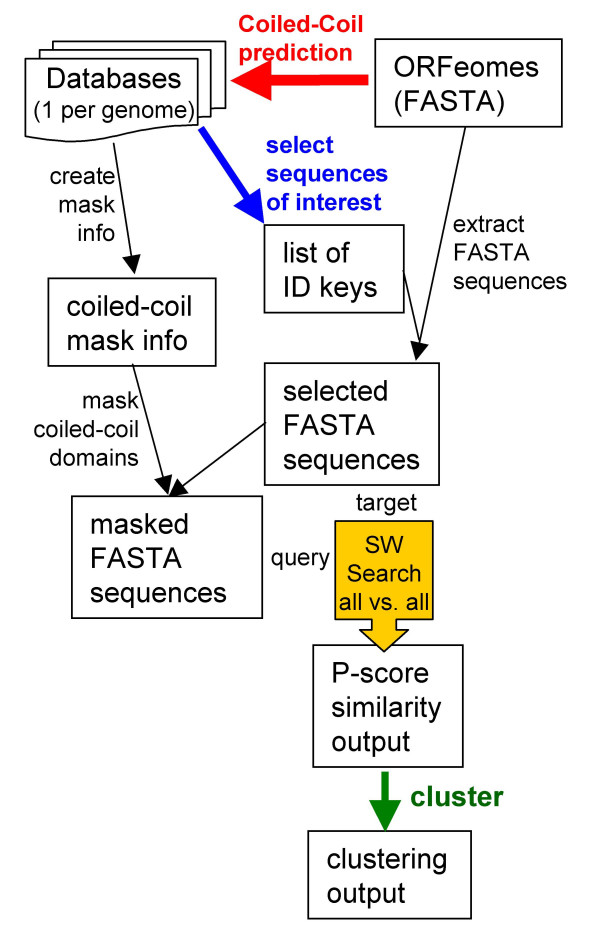
**Flowchart of sequence comparison and clustering**. Coiled-coil prediction data was generated using the program MultiCoil [42] and output processing and coiled-coil domain selection were performed as described for the ARABI-COIL database [11]. Coiled-coil prediction data was used to generate a set of sequences with coiled-coil domains masked out. The masked sequences were used as a query against unmasked sequences in an all-against-all Smith-Waterman sequence comparison (SW Search). The P-scores from this comparison were used for clustering of the output.

To prevent clustering based on the inherent coiled-coil repeat similarities, amino acids predicted to form coiled-coil domains were computationally masked out before being subjected to sequence similarity comparison (Figure 4). The clustering of the sequences with masked coiled-coil domains yielded a much more accurate grouping of known long coiled-coil protein families such as the myosins, golgins, and SMC proteins (Table 2). The largest group of long coiled-coil proteins with 58 sequences comprised the myosin motor proteins. The laminins, CASP/CDP, and nuclear lamins still exhibited the prior cluster profile, however the tropomyosin clusters did not appear after masking the coiled-coil domains. The coiled-coil coverage for many of the tropomyosins was predicted as 100% in our analysis, effectively excluding this protein family from the sequence comparison after masking.

**Table 2:** Clustering results

Annotation	# sequences	Species
Myosins	56	A.t., C.e., D.m., H.s., M.m., O.s., S.c., S.p.
SMCs	13	A.f., A.tu., B.b., B.s., C.v., H.s., M.g., M.j., M.m., M.t., S.c., S.sp., T.a.
Laminins	10	D.m., H.s., M.m.
ROCK	9	C.e., D.m., H.s., M.m.
ELKS/ERC1	7	H.s., M.m.
SLAP	5	H.s., M.m.
Kinectin	5	H.s., M.m.
Periplakin	5	H.s., M.m.
DOC1/FILIP	5	H.s.
C-Nap	5	D.m., H.s., M.m.
CASP/CDP	4	H.s., M.m.
CENP-F	4	H.s., M.m.
Lamins	4	H.s., M.m.
Hypothetical	4	C.e.
Unknown	4	A.t., O.s., S.p.
Unknown	4	A.t., O.s.

Largest clusters identified in a pilot analysis using all prokaryotic long coiled-coil proteins and eukaryotic proteins with coiled-coil domains longer than 250 amino acids or more than 60% coverage. Only clusters with four or more members are listed. ROCK, Rho-associated coiled-coil containing kinase [114]; ELKS/ERC1, Rab6-interacting protein [115], SLAP, sarcolemmal-associated protein [116]; DOC1, downregulated in ovarian cancer 1 [117]; CENP-F, centromer protein F [118]. For species name abbreviations, see Table 1.

### Clustering analysis with masked coiled-coil domains

**Figure 5:**
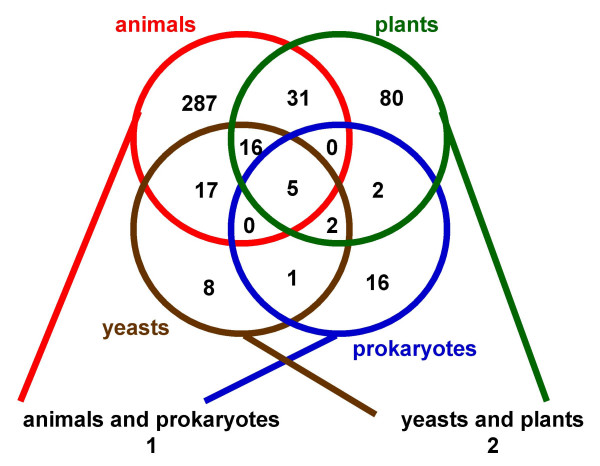
**Cluster distribution**. Clustering after Smith-Waterman comparison of sequences with coiled-coil domains masked. Numbers within the circles and overlapping sections represent numbers of clusters containing sequences from the respective kingdoms. For kingdom-specific clusters, only clusters with sequences from at least two different species were counted.

After determining the consistency of clusters formed after masking coiled-coil domains with well-known coiled-coil protein families such as the SMC proteins, myosins and kinesins, we proceeded to cluster all 3576 predicted long coiled-coil sequences from the 22 genomes. The clustering algorithm was further improved to first preclude transitively similar sequences by requiring all sequences in each cluster to satisfy the P-score threshold for all pair-wise relationships within the cluster and secondly to identify "bridge" sequences meeting these criteria for multiple clusters (see Material and Methods for details). A P-score threshold of 10e-06 was selected as the appropriate balance of sequence coverage and cluster discrimination. Table 3 gives an overview of the sequences from each species contributing to the clustering analysis using the 1.0e-06 P-score cut-off. The high number of species-specific sequences found in rice is caused by retrotransposon repeats in the rice genome containing predicted coiled-coil domains within a putative transposase ORF. Figure 5 shows the distribution of clusters among the different kingdoms. Sequence annotation including species origin provided further insight into functions and relationships among sequences in each cluster. Additional information was obtained using Conserved Domain Database searches, multiple sequence alignments, and phylogenetic tree analysis of selected clusters (see Materials and Methods).

**Table 3:** Contribution to clusters

species	ORFs total	CCs total	long CCs	species-specific	in cross-species clusters
Archaea					
T.a.	1479	29	6	1	5
M.j.	1782	54	6	2	4
A.f.	2400	65	14	6	8
S.s.	2939	77	10	8	2

gram + bacteria					
M.g.	486	21	5	3	2
M.t.	3995	70	10	7	3
B.s.	4167	144	17	10	7

gram - bacteria					
C.p.	1110	43	6	5	1
H.p.	1556	87	9	6	3
B.b.	1558	76	14	13	1
S. sp.	3164	133	25	16	9
E.c.	4356	111	16	6	10
C.v.	4400	161	19	9	10
A.tu.	5393	161	18	10	8

yeast					
S.p.	5037	303	62	25	37
S.c.	6191	344	73	35	38

plants					
A.t.	26945	1518	284	59	225
O.s.	56056	3740	997	795	202

animals					
D.m.	16196	1174	317	117	200
C.e.	22873	1234	304	144	160
M.m.	27577	1709	512	56	456
H.s.	29024	2400	855	189	666

Contribution of genomes to cross-species clusters (based on clustering using a P-score cut-off of 1.0e-06). For species name abbreviations, see Table 1.

### Coiled-coil proteins conserved between prokaryotes and eukaryotes

The SMC proteins were identified as the single major cluster of long coiled-coil proteins containing sequences from eukaryotic as well as prokaryotic genomes (see Table 4). Another group of conserved proteins with long coiled-coils comprised a number of eukaryotic Ser/Thr-kinases and a homolog from the cyanobacterium *Synechocystis *(sll0776 in Figure S1, additional file 2). However, proteins belonging to this cluster could not be found in any other prokaryotic genome.

**Table 4:** Clusters with sequences from prokaryotes and eukaryotes

**Cluster size ** **(# of sequences)**	**Max. edge ** **(P-score)**	**protein family**	**putative function/site of action**	**organisms represented**
45	4.6E-7	Structural maintenance of chromosomes 1–4	condensin, cohesin (chromatin)	A.f., A.t., A.tu., B.b., B.s., C.e., C.v., D.m., H.s., M.g., M.j., M.m., M.t., O.s., S.c., S.p, S.sp., T.a.
26	1.6E-10	Ser/Thr-kinases (DAP, DMK, GIN4, ROCK)	signal transduction	C.e., D.m., H.s., M.m., O.s., S.c., S.sp.

Numbers include "bridge" sequences qualifying for more than one cluster. Only clusters represented by at least 10 sequences are listed. DAP, death-associated protein kinase [119]; DMK, myotonic dystrophy kinase [120]; GIN4, growth inhibitory gene 4 [121]; ROCK, Rho-associated coiled-coil containing kinase [114]. For species name abbreviations, see Table 1.

A number of smaller cluster were formed containing proteins with shorter coiled-coil domains close to the cut-off for our analysis. One cluster comprised the translation initiation factor IF-2, containing the respective sequences from Drosophila, *E. coli*, mouse, rice and yeast. Another cluster with sequences conserved in prokaryotes as well as eukaryotes contained the AAA+ family ATPase ClpB/Hsp104 represented by plant, yeast and bacterial sequences. This protein functions as a protease/chaperonin in eubacteria, plants and mitochondria [35]. Two small clusters combined sequences from prokaryotes and plant genomes. One cluster comprised mitochondrial seryl-tRNA synthetases conserved in plant mitochondria as well as archaea while the second cluster comprised the PspA-like VIPP1 protein from plastids and the cyanobacterium *Synechocystis*. VIPP1 is involved in thylakoid biosynthesis in both chloroplasts as well as cyanobacteria, possibly acting in thylakoid membrane trafficking [58,59].

### Prokaryotic coiled-coil protein clusters

Prokaryotic clusters comprised membrane-bound proteins and signal transducers, as well as membrane-spanning transporters and secretion proteins such as the HlyD family [60]. The only cluster specific to prokaryotes represented by more than ten sequences in this study comprised the methyl-accepting chemotaxis proteins (MCPs; Table 5; [50]). Smaller prokaryotic clusters contained the aforementioned ABC-ATPases RAD50 and SbcC involved in DNA repair and a highly conserved group of archaeal proteins of unknown function (COG1340, represented by NP_394939 in Figure 3).

**Table 5:** Prokaryotic clusters

**Cluster size ** **(# of sequences)**	**Max. edge ** **(P-score)**	**protein family**	**putative function/site of action**	**organism**
12	<E-40	Methyl-accepting chemotaxis proteins	chemotactic sensor/signal transducer (bacterial envelope membrane)	A.f., A.tu., B.s., C.v., E.c., S.sp.

Numbers include "bridge" sequences qualifying for more than one cluster. Only clusters represented by at least 10 sequences are listed. For species name abbreviations, see Table 1.

### Eukaryotic coiled-coil protein clusters

**Figure 6:**
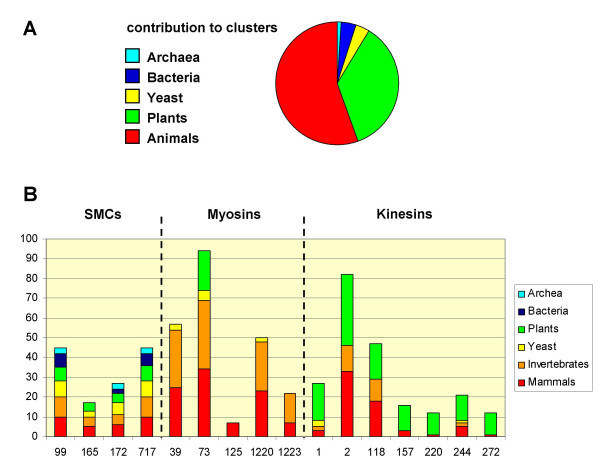
**Contribution to clusters**. Contribution of the different kingdoms to the complete sequence pool analyzed (A) and to SMC, myosin and kinesin clusters (B). Y-axis, number of sequences; X-axis, cluster IDs. Examples for characteristic protein families represented in clusters are as follows: clusters 99, 172, 717, SMC 1–4; cluster 165, SMC 5–6; clusters 39, 1220, type II myosins; cluster 125, type X myosins; cluster 1223, non-muscle myosins; clusters 1, 272, KIFCs; clusters 157, 220, PAKRPs; cluster 244, NACK, MKRPs. Proteins may qualify for two or more overlapping cluster, e.g. resulting in prokaryotic SMCs clustering with different types of diverged eukaryotic SMC proteins.

The main clusters formed by eukaryotic sequences only (Table 6) were the eukaryotic motor proteins: the actin motor myosin and the microtubule motor kinesin and the related kinesin-like calmodulin-binding protein KCBP [34,61,62]. The proteins of the SMC5 and SMC6 families formed a eukaryotic cluster instead of clustering together with the condensin/cohesin SMCs 1–4 and the prokaryotic SMC proteins in our analysis (Figure 6B). Eukaryotic RAD50 proteins clustered separately from prokaryotic RAD50s as well, indicating a higher convergence of the non-coiled-coil RAD50 ATPase domains as compared to the SMC 1–4 head and tail domains. Additional larger clusters included eukaryotic Ser/Thr-kinases and a family comprised of the Retinoblastoma-associated protein RBP95, Ring Finger Proteins 20 and 40, and yeast Bre1p [63,64,23] (Figure S2, additional file 2, and Table S15, additional file 3). Formin-related proteins associated with growing actin fibers [65,66] were found in animal/yeast and animal/plant cluster combinations. Smaller conserved eukaryotic clusters included a number of proteins involved in vesicle transport, such as a Rab6 GTPase-activating protein involved in retrograde transport [67], the golgin CASP [68] and the vesicular transport proteins P115 (see Figure S3, additional file 2), autophagy protein APG6 [69,70], and early endosome antigen (EEA1, [71]) homologs (see Figure S4, additional file 2).

**Table 6:** Eukaryotic clusters

**Cluster size ** **(# of sequences)**	**Max. edge ** **(P-score)**	**protein family**	**putative function/site of action**	**organisms represented**
94	5.4E-10	Myosin heavy chain	actin motor protein (muscle, cytoskeleton)	A.t., C.e., D.m., H.s., M.m., O.s., S.c., S.p.
27	1.5E-36	Kinesin heavy chain (KCBP, KIFC)	MT motor protein (cytoskeleton)	A.t., C.e., D.m., H.s., M.m., O.s., S.c., S.p.
21	1.1E-35	Kinesin heavy chain (Cmet/Cana, MKRPs, NACK/HINKEL)	MT motor protein (cytoskeleton)	A.t., D.m., H.s., M.m., O.s., S.p.
17	1.2E-7	Structural maintenance of chromosomes 5–6/RAD18	DNA repair (chromatin)	A.t., C.e., D.m., H.s., M.m., O.s., S.c., S.p.
12	5.0E-7	Kinases (GIN4, MET)	signal transduction	A.t., D.m., H.s., O.s., S.c.
11	<E-40	RAD50 (eukaryotic)	DNA repair	A.t., C.e., D.m., H.s., M.m., O.s., S.c., S.p.
11	1.2E-7	Retinoblastoma-associated protein, RING finger protein 20	E3 Ubi. ligase for H2B histone modification (nuclear)?	A.t., C.e., D.m., H.s., M.m., O.s., S.c, S.p.

Numbers include "bridge" sequences qualifying for more than one cluster. Only clusters represented by at least 10 sequences are listed. MKRPs, mitochondrial kinesin-related proteins [122]; NACK/HINKEL, NPK1-activating kinesin [123]; MET, [124]. For species name abbreviations, see Table 1.

### Yeast, yeast-plant, and yeast-animal coiled-coil protein clusters

Eukaryotic genomes included the baker's yeast (*Saccharomyces cerevisiae*) and fission yeast (*Schizosaccharomyces pombe*) as eukaryotic, unicellular organisms. Protein clusters found to be specific for yeast were typically small (one sequence from each yeast genome, see additional file 4) and comprised proteins involved in RNA export, such as Gle1, [72] and Mlp1 [73], the spindle assembly checkpoint protein Mad1 [74], and GRIP-domain golgins [75,76]. These proteins have known homologs in other eukaryotic proteomes, which did not cluster together with the yeast proteins, likely due to a high overall coverage with coiled-coil sequences (e.g. up to 70% coiled-coil coverage for Mlp1/Tpr, up to 74% for MAD1, and up to 75% for GRIP-golgins). Another functional group of yeast proteins were cell polarity proteins such as Spa2 and Tea1 [77,78]. Tea1 clustered together with a number of plant sequences of unknown function containing Kelch repeats [79] in combination with coiled-coil domains. Proteins that were found in clusters specific to yeasts and animals (Table 7) included the microtubule motor dynein as well as proteins involved in endocytosis and microtubule dynamics, such as intersectin, restin and cytoplasmic linker proteins (CLIP) [80]. A number of myosin subclusters, for example myosin type II, was represented only by yeast and animal but not plant sequences, consistent with previous findings [81] (see Table 7 and Figure 6B).

**Table 7:** Animal and yeast clusters

**Cluster size ** **(# of sequences)**	**Max. edge ** **(P-score)**	**protein family**	**putative function/site of action**	**organism**
57	3.3E-7	Myosin heavy chain XVIII	non-muscle and smooth muscle myosins	C.e., D.m., H.s., M.m., S.p.
50	6.5E-8	Myosin heavy chain type 2	actin motor protein (cytoskeleton)	C.e., D.m., H.s., M.m., S.c., S.p.
27	<E-40	Dynein heavy chain	MT motor (cytoskeleton, flagella)	C.e., D.m., H.s., M.m., S.c.
17	3.1E-9	Intersectins, Eps15	endocytosis	C.e., D.m., H.s., M.m., S.c., S.p.
16	9.5E-7	Png-1, IF-2, Neurofilament triplet L, Troponin T		D.m., H.s., M.m., O.s., S.c.
14	<E-40	Restin, Dynactin, CLIP proteins	linking endocytic vesicles to MTs (IF cytoskeleton), dynein activator (MTs in neurons), MT/IF associated (cytoskeleton)	C.e., D.m., H.s., M.m., S.c., S.p.
14	1.6E-15	Myosin heavy chain V	unusual myosin	C.e., D.m., H.s., M.m., S.c.
14	<E-40	DRFs	binds Rho-GTP and profilin, promotes actin polymerization (membrane cytoskeleton)	D.m., H.s., M.m., S.c.

Numbers include "bridge" sequences qualifying for more than one cluster. Only clusters represented by at least 10 sequences are listed. CLIP, cytoplasmic linker protein [80]; DRFs, diaphanous-related formins [66]; Eps15, epidermal growth factor receptor substrate 15 [125]; Png-1, postmitotic neural gene-1 [126]. For species name abbreviations, see Table 1.

### Animal coiled-coil protein clusters

From the metazoan kingdom, genomes from nematodes (*Caenorhabditis elegans*), flies (*Drosophila melanogaster*), and mammals (*Mus musculus *and *Homo sapiens*) were analyzed. Clusters that appeared to be specific to animals (Table 8) comprised a variety of proteins crosslinking cytoskeletal components with membranes, such as spectrin- and periplakin-like membrane-actin and membrane-IF crosslinkers [32,82], the plasmamembrane-scaffolding Liprins [83], the family of Merlin and Ezrin/Radixin/Moesin (ERM) proteins [84,85], and a number of Golgi- and vesicle-associated proteins. Other groups comprised centrosome-associated and mitotic spindle checkpoint proteins. Type X myosins grouped together in a metazoan cluster without plant or yeast sequences. Another animal-specific group contained coiled-coil proteins involved in structural integrity such as the extracellular scaffolding protein Laminin [26] and intermediate filament proteins including the nuclear lamins and neurofilaments [86,87]. Smaller animal-specific clusters contained protein sequences involved in cell attachment and motility, embryogenesis, spermatogenesis, and immune cell movement.

**Table 8:** Animal-specific clusters

**Cluster size ** **(# of sequences)**	**Max. edge ** **(P-score)**	**protein family**	**putative function/site of action**	**organism**
39	9.9E-7	Spectrins, Dystrophin, Nesprins	membrane/actin/MT crosslinkers (cytoskeleton)	C.e., D.m., H.s., M.m.
32	<E-40	Laminins	scaffold protein (extracellular matrix)	C.e., D.m., H.s., M.m.
25	6.7E-8	Plectin/Desmoplakin	actin/MT crosslinkers (cytoskeleton)	C.e., D.m., H.s., M.m.
22	6.5E-8	Myosin heavy chain (muscle)	actin motor protein, muscle fibers	C.e., D.m., H.s., M.m.
18	9.3E-9	Lamins	nuclear IFs	C.e., D.m., H.s., M.m.
17	5.8E-7	Neurofilament triplet L, M, Death inducer, Troponin T		C.e., D.m., M.m., H.s.
14	7.5E-7	Neurofilament triplet H, M		C.e., D.m., H.s., M.m.
14	7.5E-7	Neurofilaments (Desmin, Vimentin)	IFs	C.e., H.s., M.m.
13	2.6E-7	PP1, ASPP	apoptosis stimulating	D.m., H.s., M.m.
12	<E-40	Moesin, Ezrin, Radixin	membrane organization and stabilization (membrane cytoskeleton, cytovilli)	C.e., D.m, H.s., M.m.
11	1.7E-43	RUFY	possible role in vesicle trafficking (endosomes?)	D.m., H.s., M.m.
11	2.2E-11	Lamins	nuclear IFs	D.m., H.s., M.m.
11	3.4E-7	Restin, Dynactin, CLIP proteins	linking endocytic vesicles to MTs (IF cytoskeleton), dynein activator (MTs in neurons), MT/IF associated (cytoskeleton)	C.e., D.m., H.s., M.m.
11	3.9E-7	Png-1, Neurofilament triplet M, Troponin T		C.e., D.m., H.s., M.m.
10	<E-40	Dystrophins		C.e., D.m., H.s., M.m.
10	2.7E-8	prion-like protein		C.e., D.m.

Numbers include "bridge" sequences qualifying for more than one cluster. Only clusters represented by at least 10 sequences are listed. ASPP, apoptosis stimulating of p53 protein [127]; PP1, protein phosphatase 1 [128]; RUFY, RUN and FYVE domain containing proteins [129]. For species name abbreviations, see Table 1.

A number of the clusters containing animal sequences were limited to mammalian sequences only (Table 9). The hair fiber protein keratin was found to form the largest group of proteins specific to mammals. Other mammlian clusters comprised neurofilament proteins and crosslinkers of the actin cytoskeleton and were found to overlap with clusters containing invertebrate sequences as well. A number of smaller mammalian clusters (see additional file 5, Table S17) contained sequences of unknown function which have so far only been characterized as autoantigens or cancer antigens. Smaller clusters included the centrosomal protein Ninein, which is involved in anchoring microtubule minus ends [88], and a number of other centrosomal proteins including TACCs, C-NAP1, and Centriolin [89-91]. Other clusters included mammalian reproductive organ-specific proteins, such as sperm tail-associated proteins and mammary gland-specific proteins, vertebrate-specific transcription factors and coactivators such as the SOX proteins [92], and regulators of endothelial cell motility and clotting factors in blood vessels.

**Table 9:** Clusters with mammalian sequences only

**Cluster size ** **(# of sequences)**	**Max. edge ** **(P-score)**	**protein family**	**putative function/site of action**	**organism**
51	<E-40	Keratin type II	IF, cytoskeletal (cytokeratin), hair	H.s., M.m.
45	5.0E-7	Keratin type I (hair keratin)	IF, cuticular/hair	H.s., M.m.
32	4.2E-7	Keratin type I	IF, cytoskeletal (cytokeratin), root sheeth	H.s., M.m.
31	9.2E-7	Keratin type II	IF, cytoskeletal (cytokeratin), hair	H.s., M.m.
24	3.9E-7	Keratin type I (hair keratin)	IF, cuticular/hair	H.s., M.m.
18	1.4E-14	Neurofilaments (Desmin, Internexin, Peripherin, Vimentin)	IFs	H.s., M.m.
13	1.2E-25	Interferon-induced guanylate-binding proteins		H.s., M.m.
10	1.9E-8	Plectin, Desmoplakin, Periplakin, Envoplakin		H.s., M.m.

Numbers include "bridge" sequences qualifying for more than one cluster. Only clusters represented by at least 10 sequences are listed. See Table S16 for protein details on clusters smaller than 10. For species name abbreviations, see Table 1.

### Plant coiled-coil protein clusters

As representatives for the plant kingdom, a dicot (*Arabidopsis thaliana*) and a monocot (*Oryza sativa*) plant genome were analyzed. Clusters of long coiled-coil proteins specific to Arabidopsis and rice contained mostly sequences of so far unknown function (Table 10). The rice genome contains a large number of transposon-derived ORFs which are predicted to contain coiled-coil domains, therefore a large number of plant-specific clusters was represented by rice sequences only. These have been omitted from Table 10. Plant-specific clusters represented by both plant species analyzed included kinase interacting protein 1 (KIP1) and its relatives [93], the family of filament-like plant proteins, FPPs [94], and a cluster of putative Zinc finger transcription factors homologous to the *x1 *gene of maize [95]. Smaller clusters (see additional file 6, Table S18) included nuclear matrix constituent protein 1 (NMPC1) and relatives [96], and the chloroplast unusual positioning 1 (CHUP1) actin-interacting protein [97]. Several clusters showed overlap between the plant and animal kingdoms (Table 11). These included a number of kinesin subclusters, vesicle trafficking proteins, and Guanylate-binding proteins (Figure S5). 

**Table 10:** Plant-specific clusters

**Cluster size** **(# of sequences)**	**Max. edge ** **(P-score)**	**protein family**	**putative function/site of action**	**organism**
21	1.2E-7	Kinase-interacting protein 1-like	signal transduction	A.t., O.s.
13	7.6E-12	expressed proteins	unknown	A.t., O.s.
12	2.4E-9	FPPs	unknown	A.t., O.s.
11	2.9E-7	putative receptor kinases	signal transduction	A.t., O.s.
10	5.9E-18	Transcription factor X1-like proteins	transcription	A.t., O.s.

Numbers include "bridge" sequences qualifying for more than one cluster. Only clusters represented by at least 10 sequences are listed. See Table S17 for protein details on clusters smaller than 10. FPPs, filament-like plant proteins [94]. For species name abbreviations, see Table 1.

**Table 11:** Animal and plant clusters

**Cluster size** **(# of sequences)**	**Max. edge** **(P-score)**	**protein family**	**putative function/site of action**	**organism**
83	1.6E-38	Kinesin heavy chain (Chromokinesin, KIF3, 4)	MT motor protein (cytoskeleton), nuclear	A.t., C.e., D.m., H.s., M.m., O.s.
47	4.6E-39	Kinesin heavy chain (KIF2-4, NACK, FRA)	MT motor protein (cytoskeleton)	A.t., C.e., D.m., H.s., M.m., O.s.
29	3.0E-32	Kinesin heavy chain (KIF1, 13, 14, 16, 17)	MT motor protein (cytoskeleton), axonal transporter of synaptic vesicles	A.t., C.e., D.m., H.s., M.m., O.s.
16	5.9E-38	Kinesin heavy chain (PAKRP)	MT motor protein (cytoskeleton)	A.t., H.s., O.s.
14	2.1E-9	Plexin, Rab6 GTPase activating protein	vesicle trafficking	A.t., C.e., D.m., H.s., M.m., O.s.
13	9.4E-7	Guanylate-binding protein		A.t., H.s., M.m., O.s.
12	4.3E-27	Kinesin heavy chain (KIFC1, TH65)	MT motor protein (cytoskeleton)	A.t., H.s., O.s.
12	9.6E-35	Kinesin heavy chain (PAKRP, MKRP)	MT motor protein (cytoskeleton)	A.t., H.s., O.s.
11	3.4E-7	DRFs	binds Rho-GTP and profilin, promotes actin polymerization (membrane cytoskeleton)	A.t., C.e., H.s., M.m., O.s.

Numbers include "bridge" sequences qualifying for more than one cluster. Only clusters represented by at least 10 sequences are listed. FRA, fragile fiber [130]; PAKRP, phragmoplast-associated kinesin-related protein [131], [132]. For species name abbreviations, see Table 1.

## Discussion

### The SMC proteins are the most widely conserved coiled-coil proteins

The most widely conserved family of long coiled-coil proteins found in our study comprised the SMC proteins. Representatives from almost all species analyzed were found in this cluster, with a few exceptions such as the gram-negative bacterium *E. coli*. This is consistent with previous findings that SMC proteins are present in eukaryotes as well as all gram-positive bacteria and nearly all archaea, but only less than half of the gram-negative bacteria. It has been proposed that eukaryotic *smc *genes evolved from archaeal precursors by two consecutive gene duplications [48]. Bacteria without SMC proteins often contain an SMC-related long coiled-coil protein involved in chromosome segregation or DNA repair, such as MukB or SbcC [98,13].

### Prokaryotic coiled-coil filament proteins

While prokaryotic genomes contained less long coiled-coil proteins than eukaryotes, we found a number of so far uncharacterized long coiled-coil proteins as candidates for filament-forming prokaryotic coiled-coils. These included *Heliobacter pylori *proteins previously suggested as candidates for bacterial filament proteins [99].

### Metazoan mitotic motor proteins lack homologs in plants

The presence of a nucleus in eukaryotic cells is closely linked with the presence of a motile cytoskeleton, in particular the mitotic structures necessary to orchestrate nuclear division, and the endocytic pathway. Dolan et al. [100] proposed a list of motility proteins involved in mitotic processes as candidates for homology searches in prokaryotes to determine their evolutionary origin. We found 70% of the suggested proteins (Astrin, CENP-E, Centrin, Dynein, Dynactin, Kinesin, Kinectin, MAD, NuMA, Pericentrin) among the long coiled-coil proteins identified in our analysis, however none of them clustered together with sequences from archaea or bacteria. Interestingly, with the exception of the kinesins, we also could not find any of these proteins clustering with plant sequences. With the exception of dynein, kinesin and MAD proteins, we could not find clustering of these mitotic motility proteins with yeast sequences either.

The organization of mitotic microtubule nucleation and the composition of the nuclear envelope in plant cells differ significantly from metazoan cells [101]. One hypothesis to explain these differences is the separate development of specialized mechanisms to orchestrate open mitosis in metazoan and plant lineages, leading to the evolution of different nuclear envelope compositions, targeting mechanisms, and mitotic spindle nucleation in the plant and animal kingdoms. This model explains the absence of many metazoan mitotic motility proteins in plants as well as yeast, which undergoes closed mitosis, and suggests that this group of proteins evolved after the occurrence of open mitosis.

We could not find any plant-specific classes of coiled-coil motor proteins, but noted kinesin subclusters largely represented by plant sequences only, indicating an expansion of this group of motor proteins during plant evolution (see Figure 6B). It has been noted before that Arabidopsis contains a surprisingly large number of kinesins [102], and it has been suggested that plant-specific kinesin subfamilies might be involved in stress responses or pathogen defenses [103].

### Differences and similarities in cytoskeletal and membrane infrastructure between plants and animals

Besides the motor proteins (myosins, kinesins, dyneins), membrane tethering and vesicle transport proteins appear to be specific for eukaryotes in our clustering analysis, indicating another major class of specialized coiled-coil proteins that evolved after the formation of eukaryotic cells. It has been previously suggested that the higher content of long coiled-coil domains in metazoa compared to plants and protists indicates the presence of extensive coiled-coil matrices in animal cells and tissues [25]. One of the groups of coiled-coil proteins apparently absent in plants and yeasts are the nuclear matrix and intermediate filament proteins. No lamin sequences could be identified from the plant genomes. Other differences we noted between the plant and animal kingdoms are the lack of membrane-cytoskeleton crosslinkers and scaffolding proteins, such as spectrin-like proteins and many actin- and microtubule-associated proteins, in plant proteomes. This might indicate differences in the overall organization and networking of membrane systems and the actin and microtubule cytoskeleton in plant and animal cells.

### Differences in coiled-coil content between genomes

Earlier surveys of coiled-coil sequences in GenBank had suggested that invertebrate genomes contain more coiled-coils than vertebrates, and that animal genomes contain four times more "extended" coiled-coils (>75 amino acids) than plant genomes [25]. While we could not find such a difference for the overall coiled-coil content or the group of proteins defined as "long" coiled-coils in this study, we did note a significantly lower percentage of coiled-coils longer than 250 amino acids in yeast as well as plants compared to the animal genomes (see Figure 1). On average, the yeasts contained one third of the percentage of coiled-coils present in vertebrate genomes with domains longer than 100 and longer than 250 residues (37% and 35%, respectively), whereas invertebrates contained about two thirds (60% and 73%, respectively). The plant genomes, however, contained on average 57% of the percentage of proteins with coiled-coil domains longer than 100 amino acids, but only 22% of the coiled-coils with 250 amino acids and longer when compared to vertebrates. An interesting observation is that the human genome appears to contain more extended coiled-coil proteins than the mouse genome. Our data suggests that this is caused by the human proteome sequence set containing more unique long coiled-coil proteins without homologs in other species (see Table 3), as well as more redundant sequences in clusters (e.g. comparing counts of human versus mouse sequences in clusters listed in additional file 5, Table S17).

### Comparison with other genome-wide coiled-coil predictions

Comparable with the Arabidopsis coiled-coil protein database ARABI-COIL, this study takes a more restrictive approach to identifying coiled-coil proteins than previous genome-wide approaches to predict coiled-coil proteins [44,43]. In contrast to the older studies, our prediction criteria included a minimum coiled-coil domain length corresponding to about three heptad repeats to eliminate sequences with short stretches of predicted coiled-coils unlikely to form stable structures [11]. Using these parameters, on average about 6.4% of all proteins in the eukaryotic proteomes and about 3.5% in the prokaryotic proteomes (2.6% in archaea, 3.7% in bacteria) contained coiled-coil domains. Our results were consistent with the study of Liu and Rost [43] in that most eukaryotic genomes contained more coiled-coil proteins than prokaryotic genomes, and most bacterial genomes more than archaea. The more restrictive parameters used here resulted in predicting on average about 65–70% of the number of proteins found in those previous studies. Liu and Rost [43] further found an exceptionally high coiled-coil content in *Heliobacter pylori *with a higher percentage than *C. elegans*, and an exceptionally low coiled-coil content in *Mycobacterium tuberculosis*. Our analysis was consistent with these previous observations and resulted in 5.6% coiled-coil for *Heliobacter pylori *versus 5.4% in *C. elegans *and only 1.8% in *Mycobacterium tuberculosis*, the lowest percentage for all 22 genomes analyzed here.

### Limitations of the prediction and clustering analysis

#### Discontinuous coiled-coil domain predictions

MultiCoil provides a more stringent coiled-coil prediction than other programs such as COILS, resulting in less false positive predictions. In tests on the PDB database of solved protein structures, two-thirds of the sequences predicted by COILS did not contain coiled-coils [104]. By comparison, the programs PAIRCOIL and MultiCoil perform significantly better [42]. Occasionally, however, the increased stringency might lead to prediction of fragmented domains where continuous domains have been experimentally verified, as evident in the case of the SMC proteins (see Figure 2).

#### Selection of long coiled-coil proteins only

In this study, we focused on proteins potentially involved in structural functions. As the emphasis was placed on proteins with long or multiple coiled-coil domains, it is possible that our selection criteria resulted in the exclusion of homologs of proteins with short stretches of coiled-coil that barely qualified for the analysis. The selection criteria applied in this study have been shown to exclude 97% of the known bZIP proteins from Arabidopsis [11]. Other examples we noted are the translation initiation factor IF-2, mitochondrial and prokaryotic seryl-tRNA synthetases, and the ClpB/HSP104 family of heatshock proteins. Members of these protein families failed to meet the selection criteria for long coiled-coil domains, making it difficult to draw conclusions for these protein families from our clustering analysis. We therefore focused our attention on clusters with mainly proteins containing longer coiled-coils (>150 amino acids).

#### Effect of coiled-coil masking in the clustering analysis

When clustering sequences with long coiled-coil domain in the pilot analysis, the majority of proteins with long coiled-coil domains was grouped together in one large cluster. Many of the proteins with unknown functions in this group were annotated as "myosin-like", however only about 20% of the proteins in the cluster actually contained a myosin motor domain. In the other cases, the only similarity to myosin was the presence of a long coiled-coil domain similar to the myosin coiled-coil tail. This illustrates the ease with which long coiled-coil domains can lead to misannotations in databases with annotations based on sequence similarity searches.

Masking the coiled-coil domains before sequence comparison and clustering significantly increased the specificity of the clustering analysis, however protein sequences with high coiled-coil coverage were lost in the subsequent clustering as the masking left little to no sequence for comparison. Examples are the animal and yeast tropomyosins, many of which were predicted to contain 100% coiled-coil coverage, paramyosin, and the plant cytoskeletal protein CIP1 with more than 80% coiled-coil coverage [105].

## Conclusion

Our genome-wide identification of coiled-coil proteins and subsequent clustering provides data suggesting evolutionary conservation or uniqueness of coiled-coil proteins among 22 fully sequenced genomes. We found SMC, MukB, SbcC and Rad50 proteins to be the proteins with the longest coiled-coil domains occurring in prokaryotes, whereas eukaryotic proteomes also contained proteins with stretches of coiled-coil longer than the SMC rod domains. The high conservation of the SMC proteins and their structural relatives involved in chromosome maintenance and repair demonstrates the universal importance and conservation of DNA housekeeping mechanisms.

Long coiled-coil proteins specific to eukaryotes are predominantly involved in subcellular infrastructure maintenance and trafficking control. Table 12 gives an overview of the functional classes of long coiled-coil proteins found in our analysis and their representation in different kingdoms. The genomes of higher plants lack sequences coding for intermediate filament proteins. Many of the known mitotic spindle associated coiled-coil motor proteins in animals lack homologs in plants, consistent with the absence of a centrosomal microtubule organization center in plant cells. However, the kinesin family of microtubule motor proteins appears to have expanded during the evolution of higher plants.

**Table 12:** Summary of coiled-coil protein functions

Functional groups of coiled-coil proteins	Examples	Species represented
Chromatin organization and maintenance, chromosome segregation and DNA repair	SMCs, Rad50, SbcC, MukB, MutS	all kingdoms
Transcription and translation	Transcription and translation initiation factors, reverse transcriptase	all kingdoms
Protein trafficking and quality control	Chaperonins, secretion proteins	prokaryotes and organelles
Membrane sensors, channels and regulation of influx/export	MCPs, ion channels	prokaryotes
Sensor mechanisms and signal transduction	Receptor kinases, GTPases	eukaryotes – conserved, as well as plant and animal specific
Compartmentalization, stabilization and dynamics of membrane systems	Golgins, SNAREs, endocytic proteins	eukaryotes
Adherence	Cell adherence, extracellular matrix, intracellular adapters	eukaryotes and parasitic prokaryotes
Mechanical fiber and meshwork formation	Keratin, intermediate filaments, flagellar (e.g. sperm tail) proteins	eukaryotes, keratin only in mammals
Motility	Muscle fibers, cell motility, actin and microtubule motors	eukaryotes
Organization, stabilization and dynamics of the cytoskeleton	Actin and microtubule crosslinkers	eukaryotes, predominantly metazoa
Mitotic spindle assembly and checkpoint control	Centrosome, kinetochore and spindle pole body proteins	metazoa and yeast

The repeat nature of the coiled-coil motif makes it difficult to clearly determine sequence homology relationships between long coiled-coil proteins. Functional studies will have to reveal whether so far uncharacterized prokaryotic and plant coiled-coil proteins fulfill similar functions to metazoan counterparts.

## Methods

### Sequence data and pre-processing

Proteome sequence sets of fully sequenced genomes were downloaded from the European Bioinformatics Institute (EBI) [106] for organisms listed in Table 1, with the exception of rice. The rice proteome set was downloaded from The Institute for Genome Research (TIGR) [107]. An initial preprocessing of the FASTA files was conducted to standardize identifiers for the sequences for easier incorporation into a MySQL database.

### Coiled-coil prediction and post-processing

Prediction and selection of coiled-coil proteins was performed using the underlying schema and software systems developed to create the ARABI-COIL database [11]. In summary, the modified FASTA files were used as input for the MultiCoil application installed on the Linux Cluster of the Ohio Supercomputer Center (OSC, Columbus, OH). The MultiCoil output was post-processed using the previously described Java-based ExtractProp Suite [11] and used to establish a database of coiled-coil prediction data for each organism. The same coiled-coil selectivity criteria applied to ARABI-COIL were used to select sequences predicted to contain long or multiple coiled-coil domains. These criteria impose a minimum coiled-coil domain of 30 residues if at least three domains are present in the translated reading frame, a minimum of 50 residues if at least two domains are present, and a minimum coiled-coil length of 70 residues if only a single domain is present. Intra-domain gaps of less than 20 residues were considered contiguous for purposes of establishing domain length. The resulting data was converted to XML and used to populate MySQL databases for each genome.

### Masking of coiled-coil domains

To eliminate interference of the coiled-coil repeat motif with sequence homology analysis, coiled-coil domains were "masked" before subjecting the sequences to Smith-Waterman sequence similarity searches. Mask information was created based on the processed MultiCoil prediction data generated to populate the MySQL databases for each genome. A Java-based program was applied to the FASTA sequences selected for Smith-Waterman comparison to replace all amino acids predicted to be contained in coiled-coil domains with the letter X, effectively masking coiled-coil domains.

### Sequence similarity comparison

Smith-Waterman comparison was conducted using the TimeLogic Smith-Waterman implementation at OSC and the Blosum62 scoring matrix on all unique sequences in the combined sequences set. Sequences with masked coiled-coil domains were used as query on unmasked sequence sets as target. A P-score cut-off of 1.0e-03 was used as a threshold for selecting sequence similarity relationships. For sequences to be characterized as pair-wise similar and recovered for use in the clustering analysis, the P-score value must be less than this threshold based on the query-target Smith-Waterman comparison.

### Clustering analysis

After completing the pair-wise similarity calculation using the Smith-Waterman algorithm and extracting sequence pairs and associated P-scores, sequences were grouped using a modified version of Kruskal's minimum cost spanning tree algorithm [57]. The algorithm creates and progressively merges sub-trees of a graph in building a minimum cost spanning tree. In the algorithm, the weights of edges in the directed graph were determined by the pair-wise P-score similarity value for the sequence as a query relative to the related sequence as a target. An effective clustering can be achieved by using only P-score similarity values which are below a specified threshold, effectively creating a disconnected series of related sequences.

The clustering was tested in a pilot analysis on a combined sequence set including 527 prokaryotic long coiled-coil proteins and eukaryotic proteins containing extended coiled-coil domains of at least 250 amino acids in length or at least 60% of the protein sequence in a coiled-coil. Edges with P-scores greater than 1.0e-03 to 1.0e-15 were ignored when combining sub-trees in the algorithm. The success of the clustering was estimated by observing the clustering behavior of well-known coiled-coil protein families, such as SMC proteins and myosins. After testing the effects of masking the coiled-coil domains and optimizing cut-offs for P-scores during clustering, the complete coiled-coil sequence set containing 3576 long coiled-coil proteins from the 22 genomes was processed similarly. Different P-score thresholds were explored in efforts to increase specificity in the multi-genome sequence set while preserving comprehensive coverage. Employing Kruskal's algorithm, the 3576 sequence set resulted in 156 clusters covering 3567 sequences using a threshold of 1.0e-03, 467 clusters covering 3551 sequences using a threshold of 1.0e-6 and 850 clusters covering 3520 sequences using a threshold of 1.0e-15. (For comparison, the same algorithm yielded 490 clusters for the unmasked sequence set).

Even with the improved selectivity of the clustering demonstrated in the pilot investigation using masked coiled-coil sequences, the overall effectiveness of the resulting clustering still required refinement to achieve sufficient specificity. The use of Kruskal's algorithm for subset selection enabled transitively similar sequences to be included in specific clusters. (Transitively similar sequences are sequences in which sequence A is similar to sequence B and sequence B is similar to sequence C thereby clustering sequence A and C which would otherwise not belong to the same cluster.) One drawback of this simplified clustering is that a given sequence need only be similar to at least one other sequence in the cluster. This limitation resulted in clusters containing sequences which, while closely related to at least one other sequence in a cluster, were not closely related to every sequence within the cluster.

The algorithm was consequently improved to specifically preclude transitively similar sequences by requiring all sequences in a given cluster to satisfy the P-score threshold for all pair-wise relationships in the cluster. The new algorithm dramatically improved specificity, with the same 3576 masked sequence set generating 1213 non-overlapping clusters covering 3567 sequences, 1263 non-overlapping clusters covering 3551 sequences, and 1384 non-overlapping clusters covering 3520 sequences with the improved algorithm for the same corresponding P-score threshold values. The P-score threshold of 1.0e-06 was selected as the appropriate balance of sequence coverage and cluster discrimination required.

The interest in identifying sequences which qualified for more than one cluster and bridged multiple clusters of protein families drove a second modification of the clustering algorithm. By design, the modified Kruskal's algorithm created mutually orthogonal, non-overlapping clusters while precluding transitively similar sequences from populating the same cluster. The 'greedy' algorithm was modified to specifically identify transitively similar sequences between clusters, enabling a unique ability to identify "bridge" sequences which satisfy participation criteria in multiple clusters or protein families. The modification amounted to simply validating each sequence's individual ability to satisfy participation criteria for a cluster based on the non-overlapping cluster partitioning.

The software used to conduct the actual cluster analysis in the study is available for download at the Ohio Bioscience Library [108].

### Cluster alignments and phylogenetic tree generation

Multiple sequence alignments and phylogenetic trees were generated for clusters of interest using sequences with masked coiled-coil domains and ClustalW version 1.82 incorporating the Blossum scoring matrix [109]. Phylogenetic trees were generated using the ClustalW program with a bootstrap parameter of 10,000 and displayed using the program TreeView v.1.6.6 [110].

## List of abbreviations

CASP, CDP/cut alternatively spliced product

CC, coiled-coil

CDD, conserved domain database

CDP, CCAAT displacement protein

CENP, centromer protein

CIP1, COP1-interactive protein 1

CHUP1, chloroplast unusual positioning 1

CLIP, cytoplasmic linker protein

DAM, disheveled associated activator of morphogenesis

DIA1, Diaphanous-related formin 1

DOC1, downregulated in ovarian cancer 1

EBI, European Bioinformatics Institute

ERM, ezrin/radixin/moesin

FPPs, filament-like plant proteins

Hsp, heat shock protein

IF, intermediate filament

KCBP, kinesin-like calmodulin-binding protein

KIP1, kinase interacting protein 1

KLP, kinesin-like protein

MCP, methyl-accepting chemotaxis protein

MKRP, mitochondrial kinesin-related protein

MLP, myosin-like protein

NuMA, nuclear mitotic apparatus

ORF, open reading frame

OSC, Ohio Supercomputer Center

PAKRP, phragmoplast-associated kinesin-related protein

PP1, protein phosphatase 1

RBP, Retinoblastoma-binding protein

ROCK, Rho-associated coiled-coil containing kinase

SLAP, sarcolemmal-associated protein

SMC, structural maintenance of chromosomes

S/W, Smith-Waterman sequence comparison

TACC, transforming acidic coiled-coil

Tpr, translocated promoter region

VIPP1, vesicle-inducing plastid protein 1

XML, extensible markup language

## Authors' contributions

AR coordinated this study, analyzed the data, and prepared the manuscript. SJS participated in MultiCoil and Smith-Waterman output processing and ClustalW analysis. EAS generated MultiCoil and Smith-Waterman outputs, developed software for pre- and post-processing and coiled-coil masking, and wrote the code for the clustering algorithm. IM proposed and supervised the study and edited the manuscript. All authors read and approved the final manuscript.

## Supplementary Material

Additional file 1 **Prokaryotic coiled-coil proteins **Tables S1-S14: Protein details of all long coiled-coil proteins predicted in the prokaryotic genomes analyzed in this study. Open file with Acrobat Reader. Click here for file

Additional file 2 **Eukaryotic clusters of interest **Figures S1-S6: Phylogenetic trees based on ClustalW alignments of the sequences, displayed using TreeView v.1.6.6. Open file with Acrobat Reader. Click here for file

Additional file 3 **Sequence details for Figures S1-S6, supplement to **additional file 2. Table S15: Protein information and prediction data for sequences contained in Figures S1-S6. AGI locus numbers from TAIR are used as sequence IDs for Arabidopsis, TIGR sequence IDs are used for rice and *Synechocystis*. All other sequence IDs correspond to the EBI identifiers in the downloaded FASTA files. Max. Coil Length, longest coiled-coil domain in the protein sequence; Coil Coverage, percent of sequence predicted to be in a coiled-coil. Open file with Microsoft Excel. Click here for file

Additional file 4 **Yeast clusters **Table S18: Protein information and prediction data for sequences in yeast clusters with two species (*Saccharomyces cerevisiae *and *Schizosaccharomyces pombe*) represented. Sequence IDs correspond to the EBI identifiers in the downloaded FASTA files. Max. Coil Length, longest coiled-coil domain in the protein sequence; Coil Coverage, percent of sequence predicted to be in a coiled-coil. Open file with Microsoft Excel. Click here for file

Additional file 5 **Small mammalian clusters; supplement to **Table 9. Table S16: Protein information and prediction data for sequences in mammalian clusters with two species (mouse, human) represented and less than 10 sequences per cluster. Sequence IDs correspond to the EBI identifiers in the downloaded FASTA files. Max. Coil Length, longest coiled-coil domain in the protein sequence; Coil Coverage, percent of sequence predicted to be in a coiled-coil. Open file with Microsoft Excel. Click here for file

Additional file 6 **Small plant clusters; supplement to **Table 10. Table S17: Protein information and prediction data for sequences in plant clusters with two species (Arabidopsis, rice) represents and less than 10 sequences per cluster. AGI locus numbers from TAIR or NCBI RefSeq numbers are used as sequence IDs for Arabidopsis, TIGR sequence IDs are used for rice. Max. Coil Length, longest coiled-coil domain in the protein sequence; Coil Coverage, percent of sequence predicted to be in a coiled-coil. Open file with Microsoft Excel. Click here for file
