# Supplementary material for: Coiled-coil protein composition of 22 proteomes – differences and common themes in subcellular infrastructure and traffic control
Source: BMC Evol Biol. 2005 Nov 16;5:66. doi: 10.1186/1471-2148-5-66 (PMC1322226; doi:10.1186/1471-2148-5-66)
Supplement: Additional file 1 — Prokaryotic coiled-coil proteins Tables S1-S14: Protein details of all long coiled-coil proteins predicted in the prokaryotic genomes analyzed in this study. Open file with Acrobat Reader. [file 1471-2148-5-66-S1.pdf]

Supplementary Tables

Table S1: *Archeoglobus fulgidus* proteins with long coiled-coil domains

Acc. no.	Possible function	Protein length	CC coverage	CC domains	CC domain length	TMDs
NP_069865	SbcC/Rad50 ATPase	886	63%	1	564	0
NP_070387	SMC1	1156	48%	5	197, 117, 115, 98, 35	0
NP_069878	Methyl-accepting chemotaxis protein tlpC-2	834	27%	3	114, 79, 39	0
NP_070855	Unknown	286	69%	1	199	0
NP_069867	Methyl-accepting chemotaxis protein tlpC-1	677	28%	4	72, 54, 44, 26	0
NP_069376	Membrane protein	228	51%	1	118	1
NP_069979	Prefoldin beta (GimC beta), erpK	116	91%	1	106	0
NP_070053	Membrane protein	212	47%	1	101	1
NP_070240	Membrane protein, secreted	352	28%	3	35, 33, 31	1, SP
NP_070312	Signal-transducing histidine kinase	908	10%	3	42, 36, 20	0
NP_070386	Unknown	167	57%	1	96	0
NP_071249	Unknown	627	13%	1	87	0
NP_068888	Unknown	144	57%	2	54, 29	0
NP_070859	Seryl-tRNA synthetase	453	16%	1	73	0

CC, coiled-coil; TMDs, transmembrane domains as predicted by TMHMM; SP, signal peptide for secretion as predicted by SignalP 3.0

Table S2: *Methanococcus jannaschii* proteins with long coiled-coil domains

Acc. no.	Possible function	Protein length	CC coverage	CC domains	CC domain length	TMDs
NP_248653	SMC1	1169	54%	3	334, 271, 30	0
NP_248322	SbcC ATPase	1005	54%	3	420, 91, 36	0
NP_248250	Membrane protein	469	25%	1	121	2
NP_247982	Prefoldin beta (GimC beta), erpK protein	113	82%	1	93	0
NP_247189	v-type ATP synthase subunit E (atpE)	206	43%	1	89	0
NP_247192	Unknown	104	82%	1	86	0

CC, coiled-coil; TMDs, transmembrane domains as predicted by TMHMM; SP, signal peptide for secretion as predicted by SignalP 3.0

Table S3: *Sulfolobus solfataricus* proteins with long coiled-coil domains

Acc. no.	Possible function	Protein length	CC coverage	CC domains	CC domain length	TMDs
NP_343627	Rad50	864	53%	3	254, 159, 47	0
NP_342705	Unknown	464	59%	1	274	0
NP_343618	BPS2	587	46%	3	159, 72, 41	0
NP_342432	Unknown	298	72%	2	116, 99	0
NP_343158	Unknown	330	49%	1	162	0
NP_343226	Unknown	328	48%	1	160	0
NP_342906	Unknown	288	45%	1	131	0
NP_341927	Membrane protein, secreted	397	29%	1	116	2, SP
NP_343027	Unknown	267	41%	1	112	0
NP_342124	Seryl-tRNA synthetase (serS)	457	16%	1	74	0

CC, coiled-coil; TMDs, transmembrane domains as predicted by TMHMM; SP, signal peptide for secretion as predicted by SignalP 3.0

Table S4: *Thermoplasma acidophilum* proteins with long coiled-coil domains

Acc. no.	Possible function	Protein length	CC coverage	CC domains	CC domain length	TMDs
NP_394249	SMC	1140	46%	3	255, 237, 41	0
NP_393635	ScbC/Rad50	896	28%	3	96, 90, 67	0
NP_394939	Unknown	295	53%	2	101, 56	0
NP_394594	Prefolding beta, GimC beta	124	87%	1	108	0
NP_394260	Unknown	97	86%	1	84	0
NP_393856	Membrane protein, secreted	1379	5%	1	71	2, SP

CC, coiled-coil; TMDs, transmembrane domains as predicted by TMHMM; SP, signal peptide for secretion as predicted by SignalP 3.0

Table S5: *Mycobacterium tuberculosis* proteins with long coiled-coil domains

Acc. no.	Possible function	Protein length	CC coverage	CC domains	CC domain length	TMDs
NP_217438	SMC	1205	28%	5	158, 89, 32, 31, 30	0
NP_337306, NP_217247	Unknown	450	38%	2	139, 36	0
NP_855165	Invasion protein NlpC/P60 family, secreted	472	32%	2	77, 75	0, SP
NP_856466, NP_217313	Unknown	562	24%	1	140	0
NP_216745	Zn-ribbon protein	245	54%	1	133	0
NP_337321	PspA/IM30-like	270	40%	1	110	0
NP_855862, NP_216706	NlpC/P60 family, membrane protein, secreted	385	25%	2	55, 43	1, SP
NP_336674, NP_855818, NP_216661	antigen 84, cell division initiation protein DivIVA	260	37%	2	64, 33	0
NP_856597, NP_217443	Cell division initiation protein DivIVA	245	32%	1	79	0
NP_335940, NP_855131, NP_215960	Unknown	136	51%	1	70	0

CC, coiled-coil; TMDs, transmembrane domains as predicted by TMHMM; SP, signal peptide for secretion as predicted by SignalP 3.0

Table S6: *Bacillus subtilis* proteins with long coiled-coil domains

Acc. no.	Possible function	Protein length	CC coverage	CC domains	CC domain length	TMDs
NP_389476	SMC	1186	41%	4	274, 143, 38, 32	0
NP_390018	Transglycosylase, metalloendopeptidase	2285	15%	9	69, 52, 42, 39, 39, 30, 30, 27, 20	0
NP_388946	SbcC	1130	25%	4	120, 67, 59, 37	0
NP_391360	PspA2, NlpC/P60 family	473	43%	2	118, 86	0, SP
NP_388873	Unknown	963	13%	3	54, 45, 31	0
NP_389578	HD superfamily hydrolase, membrane protein, secreted	520	23%	1	120	1, SP
NP_391064	Membrane protein	1076	11%	2	81, 39	6
NP_390914	Membrane protein	248	47%	1	118	2
NP_390425	dnaK/HSP70	610	17%	1	105	0
NP_390839	Septation ring formation regulator ezrA, membrane protein	562	17%	3	43, 32, 25	1
NP_390736	MutS2	785	12%	1	95	0
NP_389308	Unknown	321	27%	2	62, 26	0
NP_388226	Methyl-accepting chemotaxis protein tlpC, membrane protein, secreted	573	15%	3	32, 30, 26	2, SP
NP_391067	Membrane protein	451	18%	1	84	1
NP_391584	Unknown	184	43%	1	80	0
NP_391430	two-component sensor histidine kinase degS	385	20%	1	77	0
NP_389318	Membrane-fusion protein, secreted	377	20%	1	76	1, SP

CC, coiled-coil; TMDs, transmembrane domains as predicted by TMHMM; SP, signal peptide for secretion as predicted by SignalP 3.0

Table S7: *Mycoplasma genitalium* proteins with long coiled-coil domains

Acc. no.	Possible function	Protein length	CC coverage	CC domains	CC domain length	TMDs
NP_072883	Cytadherence accessory protein hmw2	1805	40%	14	129, 80, 63, 55, 54, 54, 45, 42, 37, 36, 34, 34, 32, 30	0
NP_072997	Unknown	756	45%	3	164, 121, 60	0
NP_072965	SMC/P115	982	16%	4	45, 44, 39, 35	0
NP_072936	Unknown	340	26%	2	67, 24	0
NP_073026	ClpA/B	714	9%	1	70	0

CC, coiled-coil; TMDs, transmembrane domains as predicted by TMHMM; SP, signal peptide for secretion as predicted by SignalP 3.0

Table S8: *Agrobacterium tumefaciens* proteins with long coiled-coil domains

Acc. no.	Possible function	Protein length	CC coverage	CC domains	CC domain length	TMDs
NP_353825	SMC	1165	37%	5	153, 99, 83, 53, 51	0
NP_355392, NP_533112	Membrane protein	411	33%	1	136	1
NP_533823	Exopolysaccaride production protein, secreted	418	32%	1	134	0, SP
NP_355705	Filament-A precursor, membrane-bound metallopeptidase	486	23%	2	63, 53	0
NP_357010	Membrane protein	903	12%	3	48, 38, 28	2
NP_355330	Succinoglycan biosynthesis transport membrane protein	546	20%	2	80, 33	2
NP_356875	Flagellar motor membrane protein	343	32%	1	110	1
NP_533827	Methyl-accepting chemotaxis protein mclA, membrane protein	605	17%	2	61, 45	1
NP_356179	Multidrug resistance efflux pump, membrane protein	432	23%	1	101	1
NP_535813, NP_396376	Methyl-accepting chemotaxis protein, membrane protein	646	14%	2	55, 38	3
NP_535144, NP_355996	HlyD family secretion protein, membrane protein, secreted	354	25%	2	55, 35	1, SP
NP_354111	Multidrug resistance efflux pump, membrane protein	385	23%	1	89	1
NP_354799	HlyD family secretion protein, membrane protein	437	19%	1	87	1
NP_353067, NP_530742	two component sensor kinase	881	9%	1	85	0
NP_356240	Rhizobiocin secretion protein rspE (HlyD family), membrane protein	436	18%	1	80	1
NP_357284	HlyD family secretion protein, membrane protein	497	14%	1	73	1
NP_353742, NP_531417	ATP synthase B chain atpF, membrane protein	161	44%	1	72	1
NP_355244	HlyD family secretion protein, membrane protein, secreted	403	17%	1	70	1, SP

CC, coiled-coil; TMDs, transmembrane domains as predicted by TMHMM; SP, signal peptide for secretion as predicted by SignalP 3.0

Table S9: *Chromobacterium violaceum* proteins with long coiled-coil domains

Acc. no.	Possible function	Protein length	CC coverage	CC domains	CC domain length	TMDs
NP_903578	SMC	1162	25%	4	97, 80, 77, 44	0
NP_903023	Membrane-bound metallopeptidase, secreted	463	46%	2	131, 86	1, SP
NP_902074	KrfA	356	49%	1	177	0
NP_900531	SMC-like	936	16%	3	87, 44, 28	0
NP_902577	Methyl-accepting chemotaxis protein, membrane protein	535	20%	2	73, 73	2
NP_902143	RmuC protein family	493	23%	2	81, 33	0
NP_899739	Multidrug resistance efflux pump, membrane protein	416	24%	1	103	1
NP_901249	Two-component hybrid sensor and regulator, membrane protein	1234	8%	1	103	2
NP_902179	Methyl-accepting chemotaxis protein, membrane protein	554	17%	3	42, 31, 24	2
NP_901614	ClpA ATPase	859	11%	2	62, 33	0
NP_900018	Unknown	533	17%	1	92	0
NP_902289	Cell invasion protein, membrane protein	583	15%	1	89	2
NP_903108	Methyl-accepting chemotaxis protein	984	9%	2	54, 35	0
NP_900317	Unknown	241	34%	1	84	0
NP_903635	ClpA/B-type chaperone	875	9%	2	55, 28	0
NP_901645	NolF secretion protein, secreted	376	19%	1	73	0, SP
NP_903920	Secreted protein	109	65%	1	71	0, SP
NP_899927	Methyl-accepting chemotaxis protein, membrane protein	540	12%	1	70	2
NP_903260	Secreted protein	155	45%	1	70	0, SP

CC, coiled-coil; TMDs, transmembrane domains as predicted by TMHMM; SP, signal peptide for secretion as predicted by SignalP 3.0

Table S10: *Escherichia coli K12* proteins with long coiled-coil domains

Acc. no.	Possible function	Protein length	CC coverage	CC domains	CC domain length	TMDs
NP_415444	Chromosome partition protein MukB	1486	16%	3	116, 84, 38	0
NP_415267	TolA, membrane protein	421	52%	1	219	1
NP_415890	Side tail fiber protein GP37	1120	16%	2	126, 60	0
NP_753678	Phage shock protein A (PspA)	221	66%	1	147	0
NP_418070	Membrane protein, secreted	419	35%	2	78, 69	1, SP
NP_414931	SbcC	1048	13%	4	44, 39, 29, 28	0
NP_417944	HlyD family protein, membrane protein	355	34%	1	121	1
NP_312076, NP_417637	Initiation factor IF-2	890	13%	1	118	0
NP_417523	Membrane protein	553	20%	3	43, 41, 31	2
NP_415938	Methyl-accepting chemotaxis protein III, membrane protein	546	18%	3	43, 28, 28	2
NP_414998	Small-conductance mechanosensitive channel kefA, secreted	1120	8%	3	39, 29, 29	12, SP
NP_418583	Small-conductance mechanosensitive channel, secreted	1107	8%	3	34, 32, 29	11, SP
NP_418770	Restriction endonuclease R.EcoKI	1188	6%	1	78	0
NP_417083	ClpB	857	8%	1	75	0
NP_290557	Unknown	81	88%	1	72	0
NP_415399	Macrolide-specific efflux protein MacA, membrane protein, secreted	371	19%	1	71	1, SP

CC, coiled-coil; TMDs, transmembrane domains as predicted by TMHMM; SP, signal peptide for secretion as predicted by SignalP 3.0

Table S11: *Heliobacter pylori* proteins with long coiled-coil domains

Acc. no.	Possible function	Protein length	CC coverage	CC domains	CC domain length	TMDs
NP_206859	Unknown	284	86%	1	245	0
NP_206919	Unknown	461	33%	1	154	0
NP_207750	Zn-ribbon protein	254	52%	1	133	0
NP_207034	Unknown	433	25%	1	112	0
NP_207933	Unknown	759	13%	1	106	0
NP_207250	McrB restriction endonuclease	518	19%	1	103	0
NP_206909	Hsp70/dnaK	619	14%	3	33, 29, 29	0
NP_207249	Unknown	94	90%	1	84	0
NP_206920	Unknown	398	19%	1	79	0

CC, coiled-coil; TMDs, transmembrane domains as predicted by TMHMM; SP, signal peptide for secretion as predicted by SignalP 3.0

Table S12: *Chlamydia pneumoniae* proteins with long coiled-coil domains

Acc. no.	Possible function	Protein length	CC coverage	CC domains	CC domain length	TMDs
NP_300641, NP_224781	CHLPS incA, membrane protein	651	34%	4	122, 41, 32, 28	2
NP_876465, NP_445124	inclusion membrane protein A (incA)	390	50%	2	150, 45	2
NP_445165	Membrane protein	1537	6%	1	104	3
NP_300580, NP_224721	Zn-ribbon protein	254	38%	1	99	0
NP_445383	Membrane protein	428	22%	2	65, 33	4
NP_300070, NP_224225	Membrane protein	241	29%	1	72	2

CC, coiled-coil; TMDs, transmembrane domains as predicted by TMHMM; SP, signal peptide for secretion as predicted by SignalP 3.0

Table S13: *Borrelia burgdorferi* proteins with long coiled-coil domains

Acc. no.	Possible function	Protein length	CC coverage	CC domains	CC domain length	TMDs
NP_045547	Unknown	451	43%	1	197	0
NP_212687	Secreted protein	497	38%	2	125, 67	0, SP
NP_212646	Membrane protein	2166	8%	4	86, 37, 27, 24	1
NP_045633	Outer surface protein D (ospD), secreted	257	58%	2	113, 37	0, SP
NP_045619	Immunogenic protein	288	50%	2	96, 50	0
NP_051450	ErpQ, secreted	343	41%	2	113, 30	0, SP
NP_045621	Immunogenic protein	332	42%	2	87, 50	0
NP_051288	Outer surface protein F (ospF)	224	60%	1	135	0
NP_212179	SMC P115	819	15%	2	85, 40	0
NP_051417, NP_051200	ErpB, ErpO	378	38%	2	113	0
NP_051372	Outer surface protein ERPL, secreted	229	42%	1	98	0, SP
NP_051373	ErpM, Membrane protein, secreted	363	25%	1	92	1, SP
NP_212423	Flagellar assembly protein fliH	306	26%	1	81	0
NP_045612	Membrane protein	184	40%	1	74	1

CC, coiled-coil; TMDs, transmembrane domains as predicted by TMHMM; SP, signal peptide for secretion as predicted by SignalP 3.0

Table S14: *Synechocystis sp. PCC6803* proteins with long coiled-coil domains

Acc. no.	Possible function	Protein length	CC coverage	CC domains	CC domain length	TMDs
NP_440691	SMC1	1200	34%	5	195, 128, 42, 32, 21	0
NP_440352	SbcC	1006	27%	4	106, 79, 65, 31	0
NP_440542	DNA ligase	562	41%	2	136, 95	0
NP_441330	Membrane protein	491	42%	1	208	2
NP_439969	Membrane protein	520	35%	1	185	1
NP_440174	Unknown	535	29%	2	89, 68	0
NP_440266	Membrane protein	673	23%	2	133, 24	1
NP_440140	Membrane protein	589	23%	1	141	1
NP_441885	Secreted protein	467	28%	1	133	0, SP
NP_441776	ClpB	872	14%	1	124	0
NP_442716	Methyl-accepting chemotaxis protein I (tsr), CheD, membrane protein	891	13%	3	61, 30, 29	2
NP_442275	im30, VIPP1	267	43%	1	117	0
NP_440708	Membrane protein	514	21%	2	72, 41	1
NP_441716	Unknown	417	26%	2	57, 52	0
NP_442357	Secreted protein	501	21%	1	107	0, SP
NP_441995	Unknown	194	53%	2	52, 52	0
NP_440714	Ethylene response sensor ETR1, membrane protein	844	12%	2	71, 33	3
NP_440348	Methyl-accepting chemotaxis protein mcpA, membrane protein	869	11%	2	68, 36	2
NP_442913	ABC-type transporter, membrane protein	451	22%	1	101	1
NP_442579	Unknown	173	54%	1	95	0
NP_442655	Protein kinase pknA, membrane protein	505	18%	1	92	1
NP_440058	ATP synthase subunit b (atpG), membrane protein	143	57%	1	82	1
NP_440226	Unknown	286	27%	1	79	0
NP_441059	Unknown	265	27%	1	73	1
NP_442070	ABC-type transporter	287	24%	1	70	0

CC, coiled-coil; TMDs, transmembrane domains as predicted by TMHMM; SP, signal peptide for secretion as predicted by SignalP 3.0
