# Supplementary material for: Coiled-coil protein composition of 22 proteomes – differences and common themes in subcellular infrastructure and traffic control
Source: BMC Evol Biol. 2005 Nov 16;5:66. doi: 10.1186/1471-2148-5-66 (PMC1322226; doi:10.1186/1471-2148-5-66)
Supplement: Additional file 2 — Eukaryotic clusters of interest Figures S1-S6: Phylogenetic trees based on ClustalW alignments of the sequences, displayed using TreeView v.1.6.6. Open file with Acrobat Reader. [file 1471-2148-5-66-S2.pdf]

Supplementary Figures S1 - S6

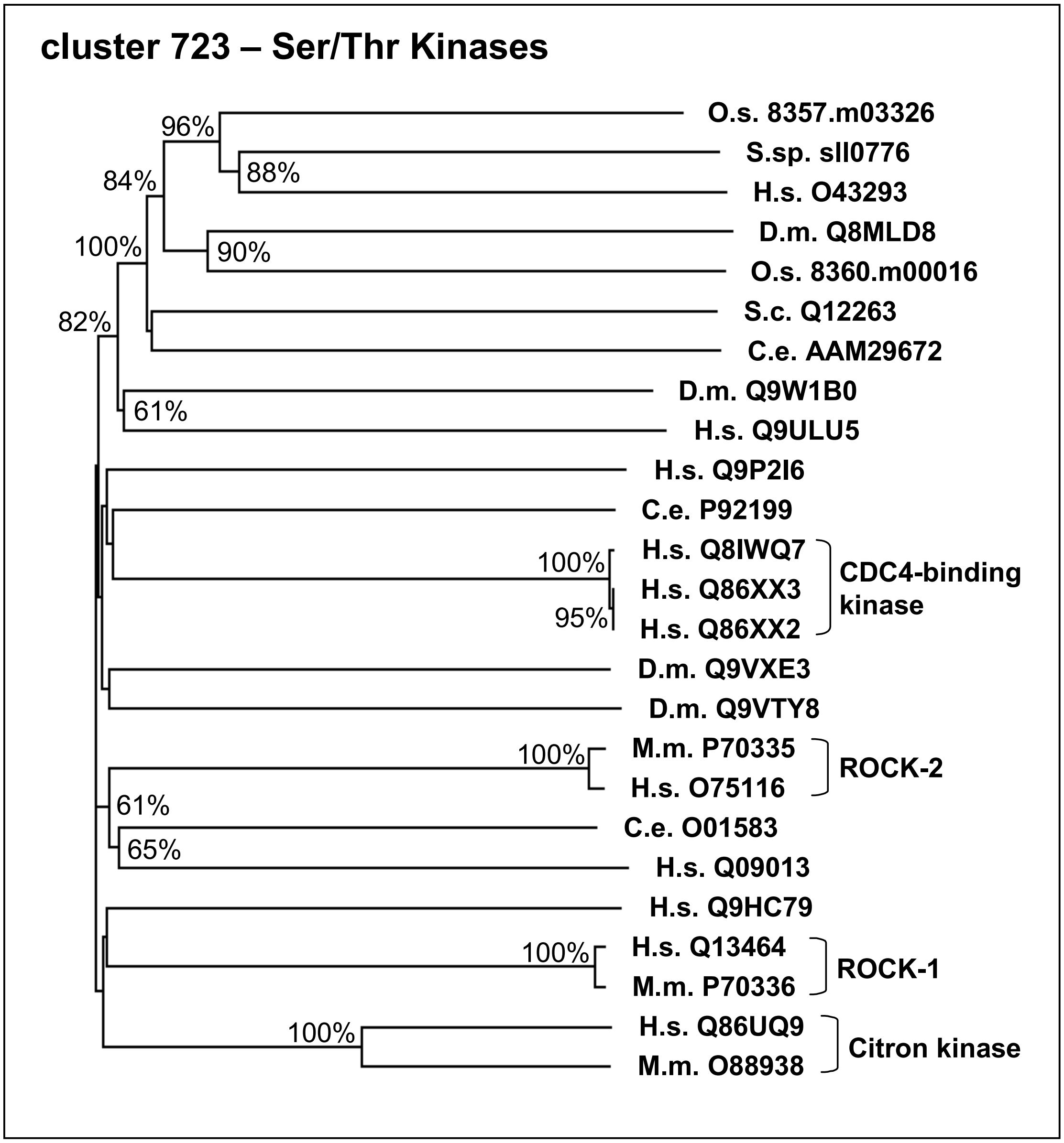

Figure S1: Phylogram of cluster 723 - Ser/Thr Kinases.

Percentages are bootstrap values (relative to 10,000). Only bootstrap values of at least 50% are shown. ROCK, Rho-associated kinase. For species name abbreviations, see Table 1; for sequence details see Table S15.

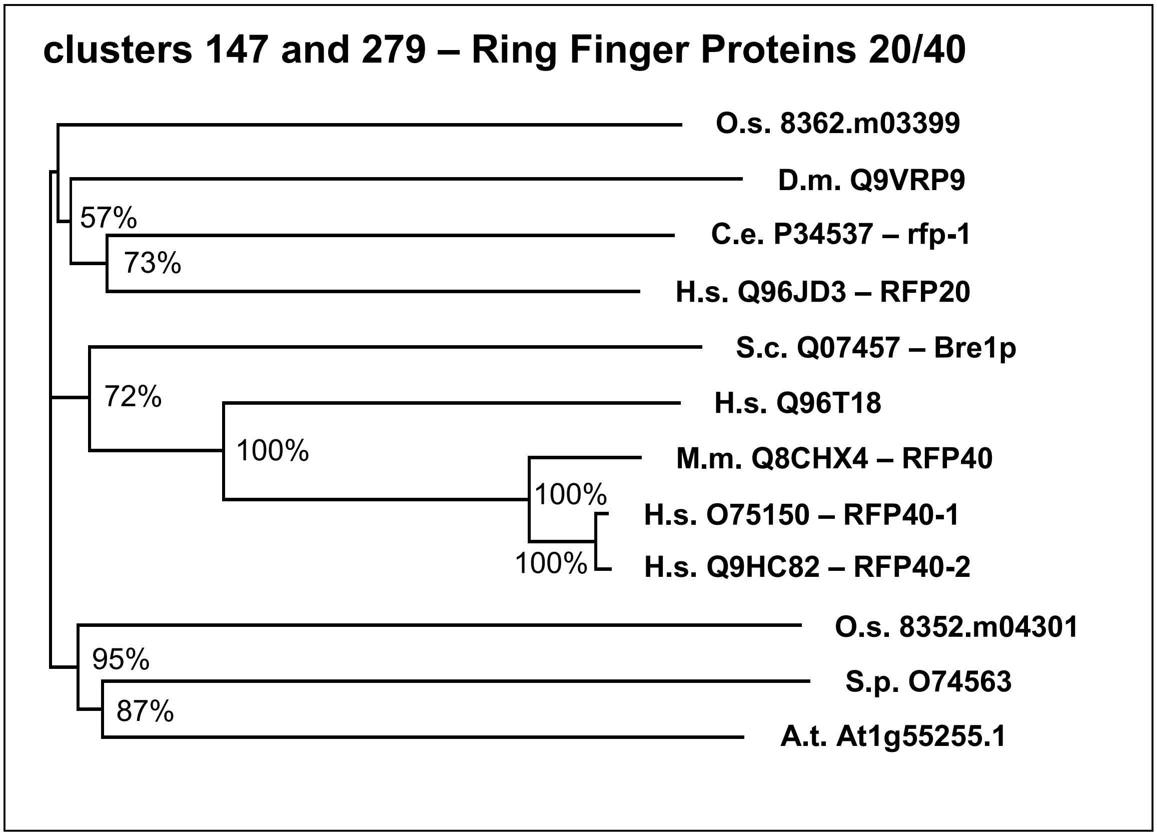

Figure S2: Phylogram of cluster 279 and 147 - Ring Finger Protein 20/40.

Percentages are bootstrap values (relative to 10,000). Only bootstrap values of at least 50% are shown. RFP, ring finger protein. For species name abbreviations, see Table 1; for sequence details see Table S15.

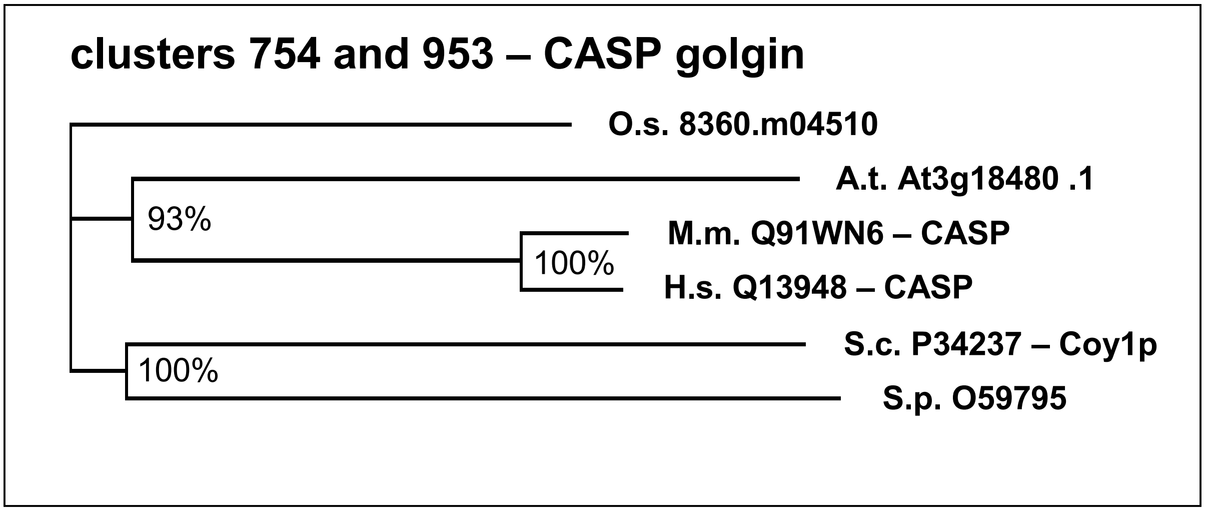

Figure S3: Phylogram of clusters 754 and 953 - CASP.

Percentages are bootstrap values (relative to 10,000). Only bootstrap values of at least 50% are shown. CASP, CDP/cut alternatively spliced product; Coy1p, CASP of yeast. For species name abbreviations, see Table 1; for sequence details see Table S15.

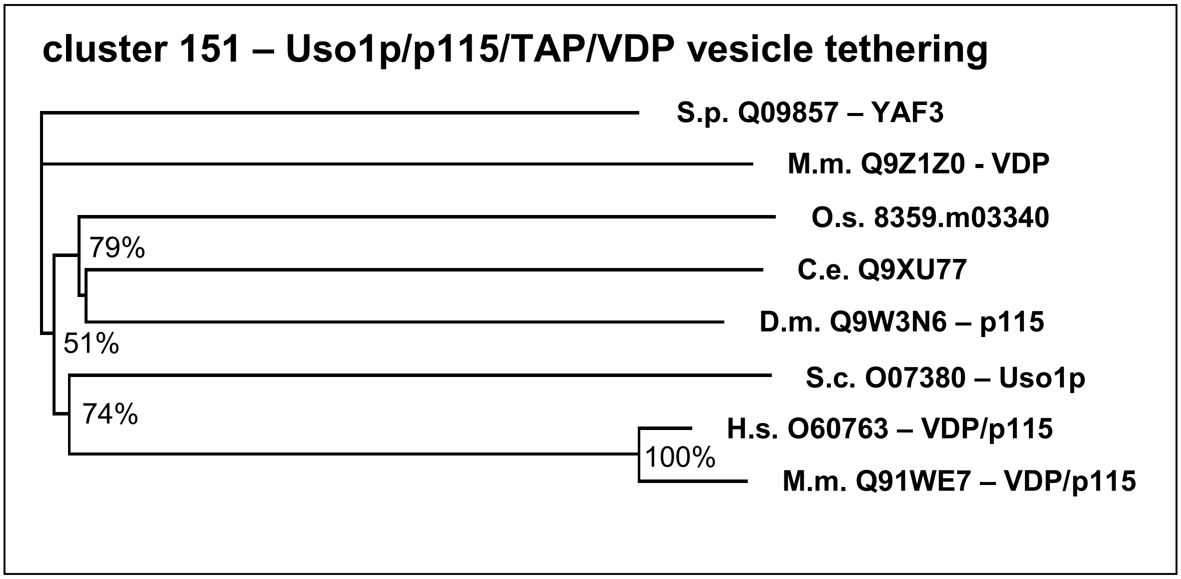

Figure S4: Phylogram of cluster 151 - VDP/P115.

Percentages are bootstrap values (relative to 10,000). Only bootstrap values of at least 50% are shown. VDP, vesicle docking protein; TAP, transcytosis-associated protein. For species name abbreviations, see Table 1; for sequence details see Table S15.

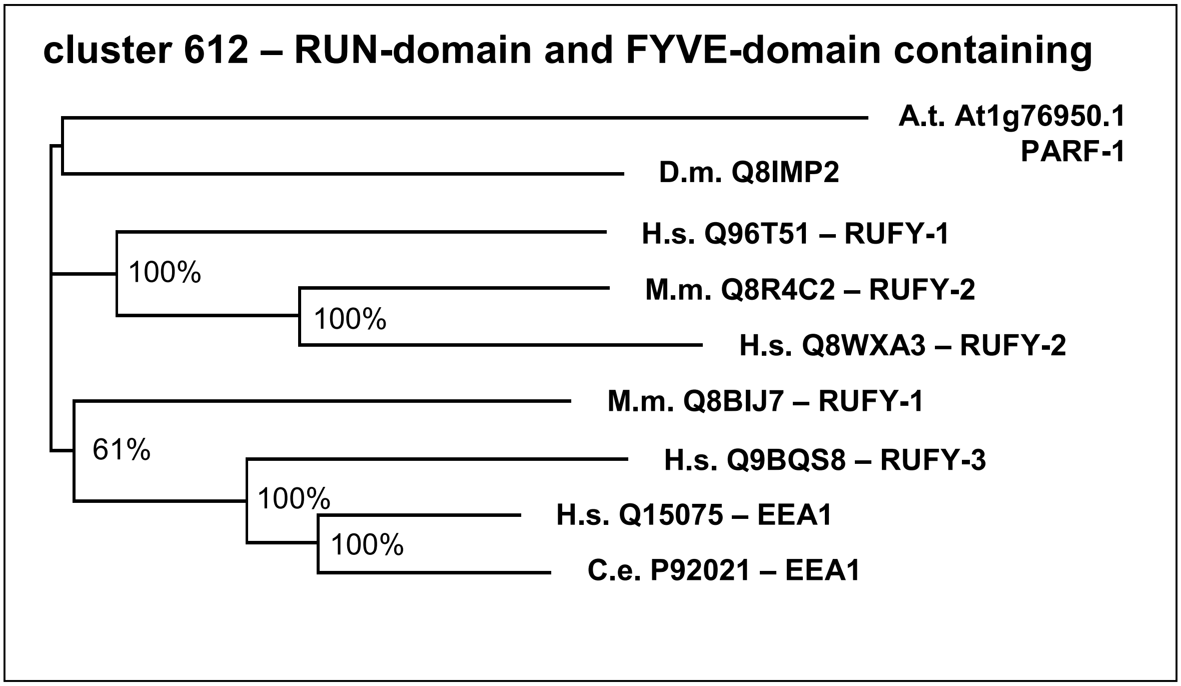

Figure S5: Phylogram of cluster 612 - RUFY.

Percentages are bootstrap values (relative to 10,000). Only bootstrap values of at least 50% are shown. EEA, early endosome antigen; PARF, PH-domain and RCC1, FYVE 1; RUFY, RUN-domain and FYVE-domain containing protein. For species name abbreviations, see Table 1; for sequence details see Table S15.

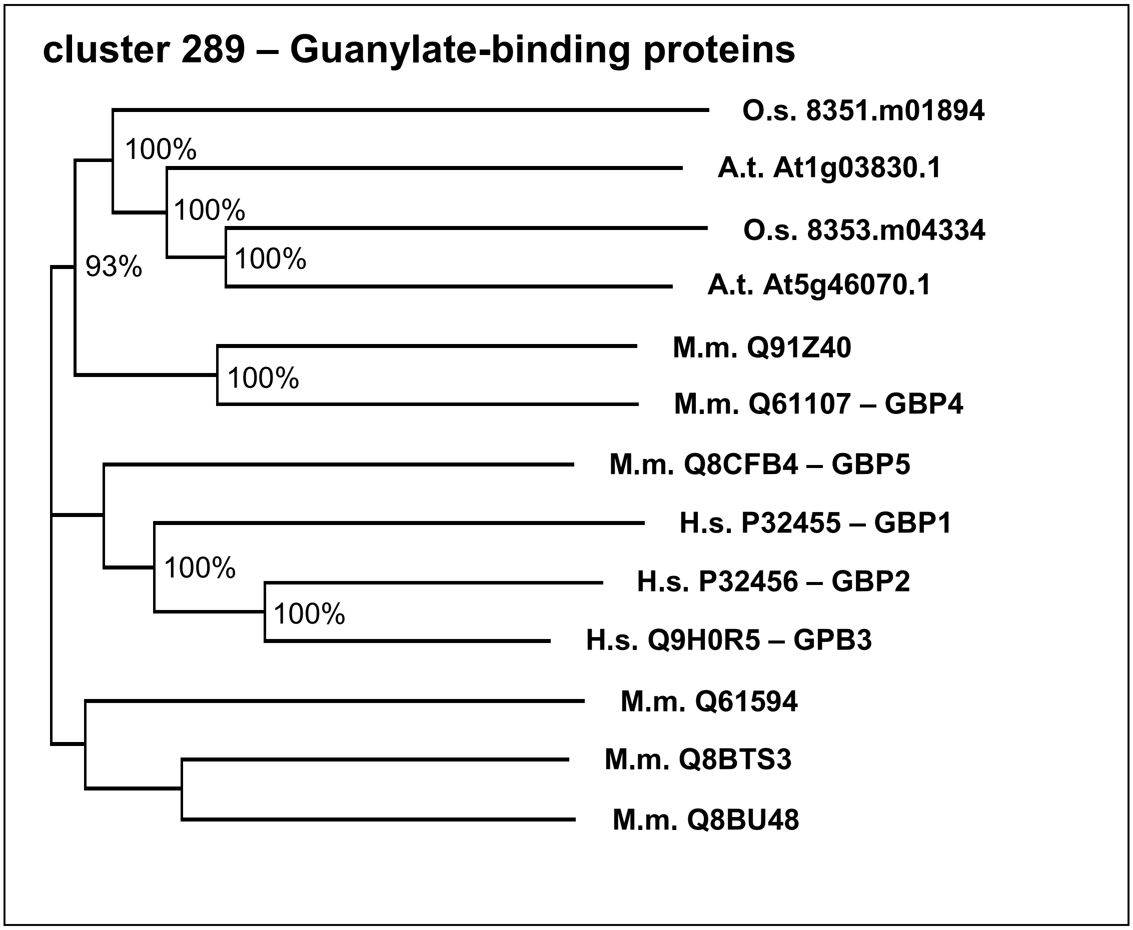

Figure S6: Phylogram of cluster 289 - Guanylate-binding proteins.

Percentages are bootstrap values (relative to 10,000). Only bootstrap values of at least 50% are shown. GBP, Guanylate-binding protein. For species name abbreviations, see Table 1; for sequence details see Table S15.
